# Exploring the Frontiers of Computational NMR: Methods, Applications, and Challenges

**DOI:** 10.1021/acs.chemrev.5c00259

**Published:** 2025-09-09

**Authors:** Susanta Das, Kenneth M. Merz

**Affiliations:** † Center for Computational Life Sciences, Lerner Research Institute, The Cleveland Clinic, Cleveland, Ohio 44195, United States; ‡ Department of Chemistry, 3078 Michigan State University, East Lansing, Michigan 48824, United States

## Abstract

Computational methods have revolutionized NMR spectroscopy, driving significant advancements in structural biology and related fields. This review focuses on recent developments in quantum chemical and machine learning approaches for computational NMR, emphasizing their role in enhancing accuracy, efficiency, and scalability. QM methods provide precise predictions of NMR parameters, enabling detailed structural characterization of diverse systems. ML techniques, leveraging extensive data sets and advanced algorithms, complement QM by efficiently automating spectral assignments, predicting chemical shifts, and analyzing complex data. Together, these approaches have transformed NMR workflows, addressing challenges in metabolomics, protein structure determination, and drug discovery. This review highlights recent progress, emerging tools, and future directions in computational NMR, underscoring its critical role in modern structural science.

## Graphical abstract

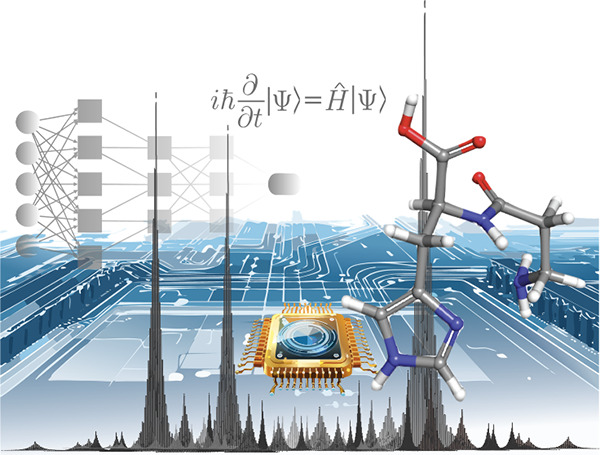

## 1 Introduction

Nuclear magnetic resonance (NMR) is a cornerstone analytical technique with broad applications across physics, chemistry, biology, and medicine, renowned for its ability to provide molecular-level insights into structure, dynamics, and interactions. Developed independently by Felix Bloch and Edward Purcell in the 1940s-a discovery that earned them the 1952 Nobel Prize in Physics-NMR has since become an indispensable tool in structural biology.^1–3^ Unlike X-ray crystallography^4–8^ and cryo-electron microscopy,^9–13^ which often require crystalline or frozen samples, NMR uniquely enables the study of biomolecules in solution under near-native conditions, capturing their conformational flexibility and dynamic behavior.^14–17^ This capability is critical for understanding the functional roles of proteins and nucleic acids, particularly in dynamic regions essential for binding, catalysis, and regulation.^18–21^ Moreover, NMR’s versatility extends beyond structural elucidation being also able to probe molecular interactions, identification of ligand-binding sites, and characterizing transient states, which makes NMR invaluable for studying complex biological processes.^22–32^

NMR is an invaluable tool in biological research, enabling insights into molecular structure, function, and dynamics across diverse systems.^33–37^ Its application in metabolomics, highlighted by Wilson and Burlingame’s 1974 study on ^13^C isotope tracers, has advanced the understanding of metabolic pathways, disease biomarkers, and cellular responses.^38^ By the 1980s, technological advancements in NMR spectrometers and magnetic field strengths solidified its role in noninvasive, high-throughput analysis of biofluids, widely applied in clinical diagnostics, pharmacology, and systems biology.^39–47^ Beyond metabolomics, NMR has contributed to protein structure determination,^48–52^ enzymatic mechanisms,^53–56^ and real-time metabolic flux monitoring,^57–59^ underpinning critical discoveries in disease progression, drug efficacy, and environmental adaptation.^60–62^ Quantitative data from NMR studies remain essential for modeling complex biochemical networks and understanding organismal physiology.^63,64^ Beyond biology, NMR plays a pivotal role in chemistry and materials science,^65–67^ supporting structural elucidation in synthetic chemistry,^33,68^ natural product isolation,^69–71^ and reaction mechanism studies.^72,73^ Solid-state NMR, as used in analyzing amorphous polymers and ceramics,^74–79^ provides detailed information on molecular arrangements, phase compositions, and interatomic distances, enabling the design of advanced materials with tailored mechanical and thermal properties.^80–82^

In the pharmaceutical industry, NMR is widely used in drug discovery and development, supporting every stage from initial screening to quality control.^83,84^ Once a tool most amenable for applicaton to small, NMR-accessible targets, its role has expanded with advancements in technology and methodology. NMR enables iterative cycles of structure determination, modeling, and chemical refinement, as demonstrated in fragment-based drug design and binding interaction studies.^85–89^ It characterizes complex drug molecules, monitors kinetics, and ensures batch-to-batch product consistency, with recent innovations like ultrafast data acquisition and automated spectral analysis accelerating lead identification. However, challenges remain, particularly in interpreting complex and overlapping spectra.^90–92^ Traditional spectral assignment is labor-intensive and expertise-dependent, limiting scalability. To address these limitations, computational methods are increasingly essential, driving advancements in algorithms and models for analyzing larger, more intricate systems. This computational shift enhances NMR’s utility in structural biology, metabolomics, and pharmaceutical research, supporting faster, more precise structural insights.

Computational approaches play a significant role in NMR spectroscopy,^93–96^ addressing challenges in structure elucidation,^97,98^ spectral interpretation,^99,100^ and molecular dynamics studies.^22,101^ Although NMR provides detailed insights into atomic environments, the interpretation of complex spectral data, particularly for large biomolecules and mixtures, often remains difficult. Computational methods enhance traditional workflows by improving accuracy and efficiency. Quantum chemical methods, including Density Functional Theory (DFT) and Coupled-Cluster (CC) calculations, enable precise prediction of NMR parameters, allowing direct comparison between experimental and simulated spectra for structure determination and verification.^102–106^ Hybrid QM/MM frameworks extend these predictive capabilities to large biomolecular systems, supporting investigations of protein–ligand interactions and material structures.^107–111^ Machine learning (ML) algorithms further advance the analysis of extensive data sets, identifying patterns in spectral data and expediting assignments. These advancements reduce dependence on experimental methods, broadening the application of computational NMR to diverse chemical and biological systems.

Quantum chemical methods, while precise, impose substantial computational costs, particularly for large or conformationally diverse molecules. Machine learning models trained on extensive compound databases mitigate this by automating peak assignments in small-molecule characterization and predicting quantum-level chemical shifts with reduced computational effort.^112–115^ Deep learning further enhances nonlinear modeling between molecular structures and spectra, improving speed and accuracy.^116–118^ Molecular dynamics (MD) simulations, integrated with NMR data, provide insights into biomolecular motions, aiding studies on protein folding, ligand binding, and enzyme catalysis.^119–122^ By interpreting parameters like nuclear Overhauser effects (NOEs) and relaxation times, MD enables flexible or disordered protein modeling.^123,124^ Computational methods integrate diverse NMR experiments with biophysical techniques such as cryo-electron microscopy and X-ray crystallography, while Bayesian inference and probabilistic models fuse heterogeneous data sets for atomic-level resolution.^125–127^ These approaches expand NMR’s applicability to biomolecules, small molecules, and materials. The growing role of computational NMR continues to reshape structural and functional analysis, enhancing efficiency, accuracy, and applicability across structural biology, metabolomics, and materials science.

Beyond these core approaches, Quantitative Structure–Activity Relationship (QSAR) modeling^128^ has proven valuable in chemoinformatics and pharmacology, predicting molecular properties from structural features and linking NMR data to functional outcomes. The integration of QSAR methods with NMR has been instrumental in studying ligand binding, drug discovery, and structural biology workflows.^129,130^

This review explores how computational methods are driving advancements in NMR, integrating quantum chemical models and ML-driven approaches to expand the field’s applicability across structural biology, chemistry, pharmacology, and materials science. By highlighting their unique contributions and synergies, we demonstrate how computational NMR is transforming molecular structure and dynamics studies. Current limitations and future opportunities are discussed to guide researchers in adopting and advancing these tools. Whether applied to metabolite characterization, protein structure elucidation, or materials research, computational NMR continues to bridge experimental data with molecular insights, offering an increasingly powerful framework for diverse scientific applications.

## 2 Quantum Chemical Methods in Computational NMR

### 2.1 Overview of DFT’s Role in Predicting Chemical Shifts and Coupling Constants

DFT has established itself as an important tool in computational NMR, offering a balance between computational efficiency and accuracy.^102,131^ By accurately modeling electronic structures, DFT excels in predicting essential NMR parameters such as chemical shifts and coupling constants, which are critical for spectral interpretation and molecular structure elucidation. Its reliability in handling a diverse range of chemical systems, from small organic molecules to complex biomolecules and materials, has made it a cornerstone method in computational spectroscopy. DFT predictions not only enhance the understanding of experimental spectra but also facilitate the characterization of novel compounds, the elucidation of complex reaction mechanisms, and the optimization of molecular properties for applications in chemistry, biology, and materials science. Furthermore, DFT methods have shown remarkable stability and precision even in challenging cases, such as transition-metal complexes with strong correlation effects, thereby broadening the scope of NMR applications. As advancements in computational power and methodologies continue to refine DFT-based approaches, its integration with experimental NMR is poised to uncover new molecular insights and accelerate progress across multiple scientific disciplines.

In contrast to other spectroscopic and analytical modalities such as mass spectrometry (MS) or chromatography, NMR spectroscopy stands out for its intrinsic computability from first principles.^132–134^ The chemical shifts and J-couplings observed in NMR are directly linked to a molecule’s electronic structure, making them highly amenable to accurate predictions using quantum chemical methods. Once these parameters are computed-typically using DFT or high-level wave function-based approaches, the entire spectrum can be constructed using density matrix formalism, capturing spin dynamics for any type of 1D or multidimensional NMR experiment. This theoretical completeness is not paralleled in MS or chromatography, where predictive modeling of fragmentation patterns or retention behavior remains largely empirical.^135,136^ The rigorous quantum mechanical foundations of NMR thus make it uniquely suitable for full spectral simulation and structural verification workflows, enabling direct comparison between computed and experimental data. Kupce and Freeman^33^ introduced an integrated NMR acquisition sequence that enables the simultaneous collection of ^13^C–^13^C INADEQUATE, multiplicity edited ^13^C–^1^H HSQC, HMBC, and standard ^13^C spectra by utilizing parallel receiver channels and cryogenically cooled probes.^137^ This approach streamlines the acquisition of multidimensional NMR data for structural characterization of small molecules. Their methodology, termed PANACEA,^138,139^ established a workflow in which structural features can be determined directly and reproducibly in a single experiment, under consistent sample conditions, without the need for fragmented data acquisition or retrospective measurements.

MS/MS fragmentation patterns, by comparison, depend on kinetic pathways that are context-dependent, often involving rearrangements, neutral losses, or charge migration phenomena that are challenging to simulate from first principles. Similarly, chromatographic retention is governed by limited characterization of molecular-surface and solvent–solute interactions, which are difficult to generalize or model mechanistically. Even modern ML-based models for LC-MS fragmentation or chromatographic retention often rely on large, system-specific training data sets and lack true first-principles interpretability. By comparison, the ability to simulate NMR spectra solely from computed chemical shifts and scalar couplings has enabled the development of comprehensive simulation platforms. For example, Bak et al. introduced SIMPSON,^140^ a general simulation package for solid-state NMR capable of modeling pulse sequences and anisotropic interactions in powdered samples. Smith et al. developed GAMMA,^141^ an object-oriented simulation library designed to represent complex spin systems in both solution and solid-state regimes. Hogben et al. advanced this further through the Spinach library,^142,143^ which incorporates large-scale Liouville space reductions and advanced relaxation modeling to simulate realistic NMR observables efficiently. These simulation frameworks demonstrate the extent to which NMR data can be reconstructed directly from quantum-derived observables, in contrast to the largely heuristic approaches required for MS and chromatography. Recent ML-enhanced protocols such as CASCADE^112^ and J-DP4 ^115,144^ further leverage computed NMR parameters to resolve stereochemistry, predict spectra, and benchmark structural candidates with minimal experimental input. The deep theoretical integration, predictive accuracy, and simulation completeness of NMR spectroscopy place it at a distinct computational advantage over other widely used analytical technologies.

### 2.2 Theoretical Background of NMR Chemical Shifts

To facilitate the analysis of NMR data independent of experimental conditions, the concept of chemical shifts, expressed on the δ-scale, was introduced.^131,145–147^ This dimensionless parameter represents the relative resonance frequency of nuclei in a sample compared to a reference standard. Mathematically, chemical shifts are defined as the ratio of the frequency difference between the sample and the reference (measured in Hz) to the operating frequency of the NMR spectrometer. Consequently, δ values are expressed in parts per million (ppm), providing a consistent and universally comparable metric for NMR data interpretation.

The chemical shift, δ, is computed using the formula established by the International Union of Pure and Applied Chemistry (IUPAC), which relies on the isotropic absolute NMR shielding constants of the sample (*σ*_*sample*_) and the reference (*σ*_*ref*_) The general equation is expressed as

$$\delta =\frac{\sigma_{ref}-\sigma_{sample}}{1-\sigma_{ref}}X10^{6}$$

However, in units of parts per million, a simplified equation is used for calculating chemical shifts:

$$\delta =\sigma_{ref}-\sigma_{sample}$$

The IUPAC equation ensures precision by correlating the intrinsic electronic environment of nuclei with their resonance frequencies, independent of the spectrometer’s field strength. Chemical shifts offer critical insights into the local electronic and chemical environments of nuclei. Variations in δ values reflect differences in electron density, molecular geometry, and intermolecular interactions, making this parameter relevant for structural elucidation, functional group identification, and dynamic studies in NMR spectroscopy.^148–155^

### 2.3 Chemical Shifts with DFT

DFT provides a robust framework for calculating these shifts^131^ by approximating molecular electronic structures through exchange-correlation functionals, as developed by Becke and Lee et al.,^156–158^ and later improved with range-separated hybrids and meta-GGAs.^159,160^ By capturing electronic shielding effects, DFT enables accurate predictions of shifts for nuclei like ^13^C, ^1^H, ^15^N, and ^17^O, essential for organic and biomolecular systems. Solvent effects, modeled using approaches like the Polarizable Continuum Model (PCM), enhance alignment with experimental data, particularly for polar molecules. Early work by Bieger et al. introduced the uncoupled perturbation treatment (UCPT) for magnetic shielding, advancing DFT’s application despite limitations in current density functional theory.^161^ These developments solidify DFT’s role in interpreting chemical shifts and supporting structural elucidation across diverse chemical systems.

### 2.4 Coupling Constants and DFT

Coupling constants (J-couplings), critical parameters in NMR spectroscopy, reveal bonding interactions mediated by electrons and molecular geometry.^162^ Ramsey’s framework decomposed J-couplings into Fermi-contact (FC), paramagnetic spin–orbit (PSO), diamagnetic spin–orbit (DSO), and spin-dipole (SD) terms, with the FC most often the dominant term for light nuclei. DFT, through coupled-perturbed Kohn–Sham (CP-KS) and finite perturbation theory (FPT), enables accurate predictions by modeling electron density and orbital effects.^163,164^ Studies by Ziegler et al.^165^ and Barone et al.^166,167^ have demonstrated DFT’s success in challenging cases, including lone-pair nuclei and dispersion-bound complexes. Solvent and relativistic effects, as noted by Mennucci et al.,^168,169^ further enhance accuracy. The interaction between nuclear magnetic moments and electrons is described by Ramsey’s nonrelativistic approach as a sum of four perturbative operators. Mathematically, the Hamiltonian (H) is expressed as

1 $$H=\underset{N<M}{\sum }\hbar^{2}\gamma_{N}\gamma_{M}I_{N}^{t}.h_{NM}^{DSO}.I_{M}+\underset{N}{\sum }\hbar \gamma_{N}I_{N}^{t}.\left(h_{N}^{FC}+h_{N}^{SD}+h_{N}^{PSO}\right)$$

In this framework, the factors *γ*_*N*_ and *γ*_*M*_ denote the gyromagnetic ratios of nuclei *N* and *M*, describing their magnetic moment per unit angular momentum. The spin operators *I*_*N*_ and *I*_*M*_ account for the nuclear spin interactions in the system. The total spin–spin coupling is decomposed into four key contributions: the Fermi-contact (FC) interaction *h*_*N*_^*FC*x^, the diamagnetic spin–orbit (DSO) interaction *h*_*NM*_^*SDO*^, the paramagnetic spin–orbit (PSO) contribution *h*_*N*_^*PSO*^, and the spin-dipole (SD) term *h*_*N*_^*SD*^. The spin–spin coupling tensor (J_NM_) is expressed as

2 $$J_{NM}=\frac{1}{h}\frac{{đ}^{2}E}{\partial I_{N}\partial I_{M}}$$

E represents the electronic energy of the system. Evaluating NMR spin–spin couplings requires determining the first-order correction to the electronic ground state, which can be derived using quantum chemical approaches. Autschbach et al. explored tensorial J-coupling properties in anisotropic environments like liquid crystals, underscoring DFT’s versatility and accuracy in diverse systems.^170–173^

### 2.5 Gauge-Including Atomic Orbitals (GIAO)

The shielding tensor (σ), a second-order property fundamental to NMR spectroscopy, has been extensively studied through quantum chemical calculations. Among the most impactful methodologies for predicting chemical shifts is the Gauge-Including Atomic Orbitals (GIAO) approach, introduced by Ditchfield^174^ and refined by Friedrich et al.^175^ within the framework of DFT. By addressing gauge invariance, GIAO enables precise modeling of molecular systems under external magnetic fields, ensuring accurate predictions of chemical shifts.^176^ Early implementations, limited by minimal basis sets and the *X*_*a*_ approximation, have evolved with modern exchange-correlation functionals and computational techniques, significantly enhancing accuracy. GIAO’s ability to incorporate the magnetic vector potential into atomic orbitals minimizes errors due to gauge origin selection, outperforming earlier methods like individual gauges for localized orbitals (IGLO)^177–180^ Its accuracy for nuclei such as ^1^H, ^13^C, ^15^N, and ^31^P has been widely demonstrated, particularly in resolving stereochemistry and structural ambiguities in organic and natural product chemistry.^181–183^

Applications of GIAO extend beyond organic systems to enzyme active sites, protein–ligand interactions, and metalloproteins where paramagnetic effects pose challenges.^184,185^ The method’s reliability is further enhanced by relativistic corrections, enabling accurate chemical shift predictions for heavy nuclei and transition metal complexes.^186,187^ Advances in linear-scaling algorithms and computational power have broadened GIAO’s scope to include biomolecules and complex materials. While challenges persist in strongly correlated systems, hybrid approaches integrating DFT with post-Hartree–Fock methods are addressing these limitations. Notably, as the importance of relativistic effects^188^ becomes increasingly recognized particularly for elements beyond the third period, the integration of relativistic corrections into GIAO frameworks represents a crucial step forward. The continuous evolution of the GIAO methodology reinforces its important role in chemical shift analysis, supporting structural elucidation across diverse chemical and biological systems.^189–192^

### 2.6 Relativistic Corrections in GIAO-Based NMR Calculations

While the GIAO methodology has been successful in predicting chemical shifts across molecular systems, its accuracy becomes limited when relativistic effects are significant. This holds for molecules containing heavy atoms, where relativistic scalar and spin–orbit contributions alter magnetic response properties. As a result, the inclusion of relativistic corrections has shifted from a refinement to a standard component of quantum chemical NMR simulations. Pioneering work by Pyykko and co-workers^188,193^ introduced a fully relativistic framework for magnetic properties, notably reinterpreting Ramsey’s theory^194,195^ of spin–spin coupling through Dirac-Hartree–Fock formalism. This early effort highlighted both the isotropic and anisotropic enhancements in the J-coupling tensor for organomercury compounds, underscoring the breakdown of nonrelativistic partitioning into contact, dipolar, and orbital terms. In subsequent reviews, Pyykko emphasized that relativistic effects arise from orbital contraction and expansion, with consequences not only for heavy nuclei but also their lighter bonded neighbors.^196^ Such perturbations of the electron density directly influence shielding and spin–spin interactions in both direct and indirect ways.

The total magnetic shielding tensor, σ, in the relativistic regime, is commonly decomposed as

3 $$\sigma =\sigma_{non-rel}+\Delta \sigma_{scalar}+\Delta \sigma_{SO}$$

where *σ*_*non*_ _–*rel*_ is the nonrelativistic shielding tensor typically obtained using GIAO within Hartree–Fock or DFT, Δ*σ*_*scalar*_ accounts for scalar-relativistic corrections such as mass-velocity and Darwin terms, and Δ*σ*_*SO*_ represents contributions from spin–orbit coupling. These corrections reflect modifications to the electronic structure induced by the relativistic increase in nuclear potential, becoming significant especially for elements beyond the third period. To treat relativistic effects within an efficient and chemically accurate framework, several approximate Hamiltonians have been developed. Among them, the Zeroth-Order Regular Approximation (ZORA)^197^ is widely used and expresses the relativistic Hamiltonian as

4 $${Ĥ}_{ZORA}=V+\frac{1}{2}\left[\mathrm{p⃗}.\frac{c^{2}}{2c^{2}-V}.\mathrm{p⃗}\right]$$

where *V* is the scalar potential, *p⃗* is the momentum operator, and *c* is the speed of light. This formulation results from a first-order expansion of the relativistic kinetic energy operator, capturing dominant scalar relativistic corrections without explicitly including spin–orbit terms. It has been successfully applied in systems such as halosilanes, where the presence of heavy halogens like iodine induces large shifts in the NMR parameters of the central silicon atom.

The Douglas–Kroll–Hess (DKH) transformation offers a hierarchy of scalar relativistic approximations by block-diagonalizing the Dirac Hamiltonian. Commonly used levels such as DKH2 or DKH3 provide a balance between accuracy and computational cost and are frequently implemented in conjunction with GIAO methods.^198,199^ The integration of the DKH transformation with GIAO has been established as a robust approach for the calculation of NMR shielding tensors, particularly for systems in which relativistic effects are pronounced.^200^ In this methodology, the DKH transformation is employed to systematically decouple the Dirac Hamiltonian, yielding a quasi-relativistic Hamiltonian that retains the essential scalar-relativistic corrections required for accurate electronic structure descriptions of heavy-element compounds. Subsequently, the GIAO technique is applied to ensure gauge-origin invariance in the evaluation of magnetic properties, thereby eliminating artifacts associated with the choice of origin in the presence of an external magnetic field. Through this combined framework, relativistic corrections are incorporated variationally, and the shielding tensor is obtained by analytical differentiation of the electronic energy with respect to the external magnetic field and the nuclear magnetic moment. The resulting approach has enabled the reliable prediction of NMR shielding constants for transition-metal and lanthanide complexes, where both electron correlation and relativistic effects are significant contributors to the observed chemical shifts.

The nuclear magnetic shielding tensor can be expressed as the second-order mixed derivative of the total electronic energy *E* with respect to the external magnetic field *B⃗* and the nuclear magnetic moment *μ⃗*_*A*_:

5 $$\sigma_{AB}={\frac{\partial^{2}E}{\partial \mu_{A}\partial B_{B}}|}_{\mathrm{B⃗}=0}$$

Through this response-theoretic formulation, relativistic corrections can be incorporated directly via the energy functional or through response terms, depending on the method employed. Importantly, the exact two-component (X2C) approach^201^ enables simultaneous inclusion of both scalar and spin–orbit effects, yielding results comparable to four-component Dirac methods but with substantially reduced computational demand. Studies involving nuclei such as ^195^Pt, ^199^Hg, and ^103^Rh consistently show that relativistic effects can alter computed chemical shifts by hundreds of ppm, further validating the necessity of these corrections.

The integration of relativistic treatments into GIAO-based NMR calculations has expanded the applicability of computational spectroscopy to systems where relativistic effects influence magnetic response properties.^202^ These methods support consistent predictions across the periodic table, including organometallics, heavy-element compounds, and bioinorganic systems, where nonrelativistic approaches lead to systematic errors. The incorporation of these corrections into GIAO frameworks has become a foundational element of predictive NMR protocols, allowing for accurate evaluation of shielding constants and coupling interactions in molecules containing both light and heavy elements.

### 2.7 Coupled-Cluster and Other High-Level Methods

Coupled-Cluster (CC) theory, introduced by Cizek^203,204^ and advanced by Bartlett and collaborators,^205–207^ is renowned for its exceptional accuracy in electronic structure calculations and its pivotal role in computational NMR.^208–211^ By expanding the wave function as a linear combination of Slater determinants, CC methods efficiently capture correlation energy through connected excitations, as demonstrated by Shavitt and Stanton. Widely used models such as CCSD and CCSD­(T) accurately capture electron correlation effects, with CCSDT and CCSDTQ extending this framework to include higher-order excitations, as highlighted by Crawford and Piecuch.^212,213^ The equation-of-motion CCSD (EOM-CCSD) approach, introduced by Perera and Bartlett,^214^ facilitates accurate spin–spin coupling constant predictions, particularly for systems with delocalized electron densities and shielding effects.^215^ Stanton and Gauss developed the analytic second-derivative implementation of CCSD, enabling precise computation of indirect spin–spin coupling constants.^216–218^ These computationally intensive methods, though limited to small molecules, are invaluable for benchmarking other approaches, validating experimental data, and resolving complex spectra, as shown by Kállay, Gauss, and Auer.^219,220^

Coupled-Cluster methods have proven instrumental in studying unusual bonding situations, particularly in heavy-element systems where relativistic effects influence NMR observables. Gauss and co-workers extended the CCSDTQ analytical derivative approach to explore bonding intricacies in organometallic and transition metal complexes,^221^ while Neese and colleagues developed parallel algorithms and the DLPNO–CCSD­(T) method,^222,223^ significantly reducing computational costs and enabling CC-level accuracy for larger systems. The integration of CC methods with molecular dynamics (MD) simulations, as demonstrated by Hättig and Piecuch, has allowed dynamic modeling of conformational flexibility, solvent effects, and thermal motion, providing NMR parameters that align closely with experimental data.^224,225^ Beyond CC theory, the second-order polarization propagator approximation (SOPPA), introduced by Geertsen and Oddershede,^226^ offers efficient spin–spin coupling constant calculations, particularly for larger molecules. Variants such as SOPPA­(CCSD), employed by Wigglesworth^227,228^ and Åstrand,^229^ address vibrational effects and provide a complementary alternative to CC methods when computational efficiency is critical. These advancements highlight the evolving versatility and accessibility of high-level quantum chemical methods in computational NMR.

Multiconfigurational self-consistent field (MCSCF) methods, such as the complete active space SCF (CASSCF) approach, introduced by Vahtras et al.,^230^ are relevant for systems with substantial static correlation, achieving accurate results for small molecules like acetylene (C_2_H_2_).^231^ The restricted active space SCF (RASSCF) extends MCSCF capabilities^232,233^ by incorporating partial dynamical correlation, as demonstrated by Jordan et al., who combined it with vibrational averaging to study spin–spin coupling in cyanamide derivatives.^234^ Full configuration interaction (FCI) theory,^235^ the benchmark for QM calculations, inherently accounts for all electronic excitations and has been invaluable for small systems, as highlighted in studies by Crawford et al.^236^ However, its steep computational scaling limits practical applications, leading to approximations such as the DLPNO–CCSD­(T) framework,^223^ which extends FCI-level precision to larger molecules. Together, these high-level methods, supported by vibrational corrections, continue to advance computational NMR, balancing accuracy and scalability in complex systems.

### 2.8 Applications and Challenges of QM Methods in Computational NMR

Quantum chemical methods are useful in interpreting and predicting NMR spectra, facilitating structural determination and molecular dynamics studies. A key application is structure determination, where quantum methods correlate experimental NMR parameters with molecular geometry. Predictions of chemical shifts and coupling constants are vital for verifying or refining molecular structures, particularly in natural product chemistry, where DFT-based ^1^H and ^13^C shift predictions help resolve ambiguities in complex spectral data.

The application of QM methods has been used for spectral assignments where experimental NMR data are insufficient for unambiguous interpretation. Facelli employed DFT calculations to revise the ^13^C and ^15^N chemical shifts in methyl bacteriopheophorbide-a (MBPheo-a) and methyl bacteriochlorophyll-a (BChl-a).^237^ Using optimized molecular geometries, the calculated ^13^C chemical shifts showed significantly better alignment with experimental data, enabling the reassignment of pyrrolic ring carbons separated by over 5 ppm. This highlighted the importance of geometry optimization, including proton positions, in improving agreement between computed and experimental values. However, ^15^N shifts exhibited poorer agreement, emphasizing the need to incorporate environmental effects, such as solvent interactions. Facelli’s study demonstrated the sensitivity of computed shifts to geometry and provided critical evidence for spectral reassignment, advancing the use of quantum chemical methods in NMR analysis.

Since the application of perturbation theory to NMR properties by Ramsey over 70 years ago,^238^ QM methods for the computation of NMR parameters have seen significant advances. Numerous reviews highlighting these advances have covered the theoretical and practical aspects of these improvements. Kaupp, Buhl, and Malkin edited a comprehensive book on the calculation of NMR and EPR parameters, providing a foundation for future work.^239^ Casabianca and de Dios published a detailed review in 2008,^240^ focusing on *ab initio* NMR chemical shift calculations. Bryce and Wasylishen^241^ contributed an extensive review of NMR parameter computations using *ab initio* methods, while Oldfield reviewed^242^ chemical shielding calculations on proteins, peptides, and amino acids, highlighting their relevance in structural biology. Facelli’s^243^ review provided insights into the applications of chemical shift calculations across various systems. Key contributions to the development of quantum chemical methods for NMR parameter calculations were reviewed by Gauss et al.,^244^ Fukui et al.,^245^ Helgaker et al.,^104^ Aucar et al.,^246^ Autschbach et al.,^247^ Rusakov et al.,^248^ Facelli,^243^ and others.^102,249,250^ These works have collectively established the computational framework for NMR studies. The early implementations of coupled-cluster theory for NMR parameter calculations, including CCSDT, CCSD­(T), and CC3, were pioneered by Auer and Gauss,^219^ with later improvements extending computations to larger systems.

Significant advancements have also been made in addressing solvent and electrostatic effects on chemical shifts. Benzi et al.^251^ and Bagno et al.^252,253^ incorporated solvent effects, while Oldfield^254,255^ applied electrostatic effects to protein structure determination. Tossell^256^ used cluster models to compute shielding values for crystals, and Sebastiani^257^ reviewed chemical shift calculations in condensed phases. Relativistic effects, crucial for heavy-element compounds, have been explored by Pyykkö and Nakatsuji et al., with applications including spin–orbit coupling and finite-perturbation theory.^188,258^ Applications of QM methods extend to the determination of relative stereochemistry, regioisomer assignments, and structural elucidation of peptides, proteins, and nucleic acids. Notably, Oldfield pioneered the use of computed chemical shift data for structural information on peptides and proteins.^242^ Bryce and Sward summarized quadrupolar halogen studies using solid-state NMR.^259–261^ Tantillo et al.^249^ reviewed the use of *ab initio* and DFT methods for chemical shift prediction, emphasizing their application to complex molecular systems. Renslow et al.^262^ highlighted the role of QM computations in metabolomics, discussing their impact on the omics community for structural predictions. Benchmarking studies on the accuracy of chemical shift predictions, conducted by various researchers, have underscored the need for reliable computational methods. Extensive summaries of advancements in evaluating chemical shielding values have been provided by Jameson and de Dios,^263,264^ further emphasizing the field’s depth and breadth. This wealth of literature reflects the importance of computational NMR in addressing structural challenges and advancing our understanding of molecular systems.

Sychrovský and co-workers extensively analyzed scalar spin–spin coupling constants (SSCCs) in guanosine and deoxyguanosine using DFT, focusing on their geometrical and solvent-dependent behaviors.^265^ The study addressed limitations of empirical Karplus equations, proposing theoretical parametrization to incorporate detailed geometry and solvent effects for improved accuracy. Using CP-DFT with the B3LYP functional, relationships between sugar pucker, glycosidic torsion angles, and SSCCs were established. 1J­(C1̀-H1̀) couplings ranged from 154 to 170 Hz, with maxima near anti conformations at X ≈ 180°, consistent with experimental values reported by Kline and Varani and Tinoco.^266–270^ Variations under solvation and solute–solvent interactions were modeled, enhancing structural interpretations and refining empirical models for nucleosides. Sychrovský’s work highlights quantum chemical calculations as a key tool for refining NMR interpretation and predicting structural details in nucleic acids.^271,272^

The relationships between ^31^P chemical-shift tensors and backbone conformation in nucleic acids were examined by Přecechtělová et al., focusing on dimethylphosphate (dmp) and dinucleoside-3′,5′-monophosphates.^273,274^ DFT calculations, both *in vacuo* and with explicit solvation, revealed significant reductions in chemical shift anisotropy (CSA) due to solvation, with variations up to 30 ppm in principal components δ_22_ and δ_33_ linked to torsion angles α and ζ. The isotropic shift (*δ*_*iso*_) for the gg conformation in solvent aligned with experimental data, affirming dmp as a reliable model. Similarly, Van Mourik and Dingley utilized DFT to study hydrogen-bonding regions in guanine quartets (G-quartets), revealing dependencies of trans-hydrogen-bond couplings and isotropic shifts on bond length and geometry.^275,276^ Li^+^, Na^+^, and K^+^ ions showed distinct preferences for stabilization positions, with K^+^ exhibiting higher energy barriers for channel transport, linked to DNA quadruplex dynamics. These studies demonstrate the power of DFT in unraveling the structural and dynamic properties of nucleic acids, offering insights into conformational effects and mechanisms of stabilization.

The structural reassignment of serlyticin-A, initially isolated from Serratia ureilytica and previously identified for its antioxidative and antiproliferative properties, demonstrates the key role of advanced methods in helping to resolve inaccuracies in natural product characterization. In a recent study, Ka Yi Tsui et al.^277^ utilized QM calculations to predict NMR chemical shifts, revealing significant discrepancies between the reported experimental data and the originally proposed structure (structure 1). Structural optimizations and frequency calculations using Gaussian at B3LYP/6-31+G­(d,p)^157,278^ and PCM­(MeOH)-B3LYP/6-31+G­(d,p)^279–281^ indicated the thermodynamic instability of the initial assignment. This led to the proposal of alternative structures, such as 3-indolylacetohydroxamic acid (structure 4), which were subsequently validated experimentally. As summarized in Figure 1, deviations between calculated and experimental ^13^C and ^1^H chemical shifts were substantially reduced for structure 4, falling within acceptable limits (≤6 ppm for ^13^C and ≤ 0.3 ppm for ^1^H), thus supporting the reassignment. For unsymmetrical dimers, averaged shifts from the two monomers were reported. Another recent study by Armando Navarro-Vázquez (2024)^282^ focused on the structural revision of marinoaziridines A and B, originally proposed as aziridine alkaloids. Using a combination of computer-assisted structural elucidation (CASE)^126^ and DFT-based chemical shift predictions, including GIAO/PBE0/def2-TZVP with ORCA^222^ and CPCM solvation models,^279,283^ the structures were reassigned as pyrroloquinoline alkaloids. This study emphasized the utility of tailored NMR data analysis in structural elucidation. Both works highlight the impact of advanced techniques in addressing structural misassignments, enhancing the accuracy of natural product characterization, and reinforcing their important role in modern structural studies.

**Figure 1:**
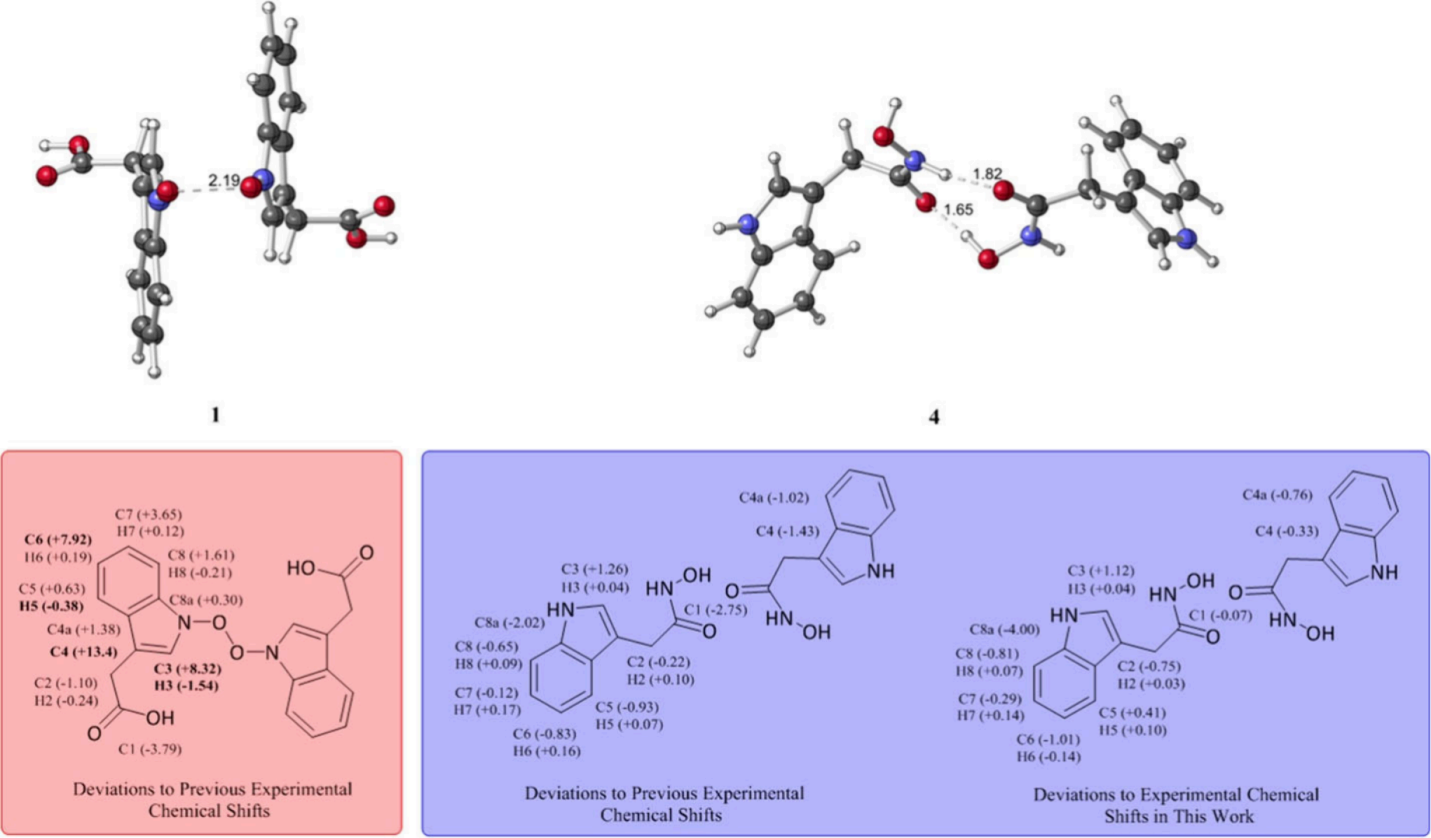
Comparison of calculated and experimental NMR chemical shifts. Left: Deviations between calculated shifts for compound 1 and its experimental values. Center: Deviations between calculated shifts for compound 4 and previously reported experimental data for serlyticin-A. Right: Deviations between calculated and newly determined experimental shifts for compound 4. Deviations are considered acceptable if within 6 ppm for ^13^C and 0.3 ppm for ^1^H, values exceeding these thresholds are highlighted in bold. For unsymmetrical dimers, chemical shifts were averaged across monomer units. Reproduced with permission from reference 277. Copyright (2019) American Chemical Society.

The accurate treatment of solvation has been established as a crucial factor in the quantum chemical prediction of NMR chemical shifts.^284,285^ Solvent molecules alter the electronic environment of nuclei through dielectric polarization, hydrogen bonding, and short-range specific interactions, which can strongly perturb nuclear shielding, particularly for labile nuclei such as ^1^H, ^15^N, and ^31^P. Implicit solvent models including the Polarizable Continuum Model (PCM),^286,287^ Integral Equation Formalism PCM (IEF-PCM),^288^ and the Conductor-like Screening Model (COSMO)^289^ are widely employed for their computational efficiency and ability to approximate bulk electrostatic effects. However, such continuum approaches are often insufficient for capturing directional interactions and dynamic solvent fluctuations near polar functional groups. To address these limitations, explicit solvent models^290^ and hybrid QM/MM schemes,^291^ have been increasingly adopted to account for local solvent structure and thermal averaging. These strategies enable a more realistic description of solute–solvent interactions and are especially beneficial in systems where hydrogen bonding and conformational flexibility significantly influence the observed chemical shifts.

A recent computational study by Da Silva et al. (2025)^292^ has further underscored the critical role of solvent modeling in the accurate prediction of NMR chemical shifts for complex molecular systems. Using azithromycin (AZM), an antibiotic characterized by significant conformational flexibility and multiple polar functional groups as a test system, QM calculations were performed by combining DFT with PCM solvent model to evaluate the influence of solvation on both relative conformer energies and NMR parameters. In addition to continuum solvation, explicit solvent molecules (chloroform, water, and DMSO) were incrementally introduced around key hydrogen bonding sites. It was shown that this hybrid approach significantly improved the agreement between theoretical and experimental NMR chemical shifts, particularly for ^1^H nuclei involved in hydrogen bonding, such as hydroxyl protons. The explicit solvent models were found to better capture local electrostatic interactions and directional hydrogen-bonding patterns that are otherwise neglected in continuum-only approaches. The use of just five explicit solvent molecules placed at strategic positions, in combination with PCM, was shown to be sufficient for achieving chemically accurate predictions at a manageable computational cost. These results provide a practical guideline for balancing efficiency and accuracy in solution-phase NMR calculations. To illustrate this point, the effect of explicit solvation on predicted NMR accuracy and conformer energetics is summarized in Figure 2. This study reinforces that the inclusion of explicit solvent molecules in conjunction with implicit models is often necessary for the reliable computational elucidation of molecular structure, particularly in natural products and biomolecules with labile protons and polar functional groups.

**Figure 2:**
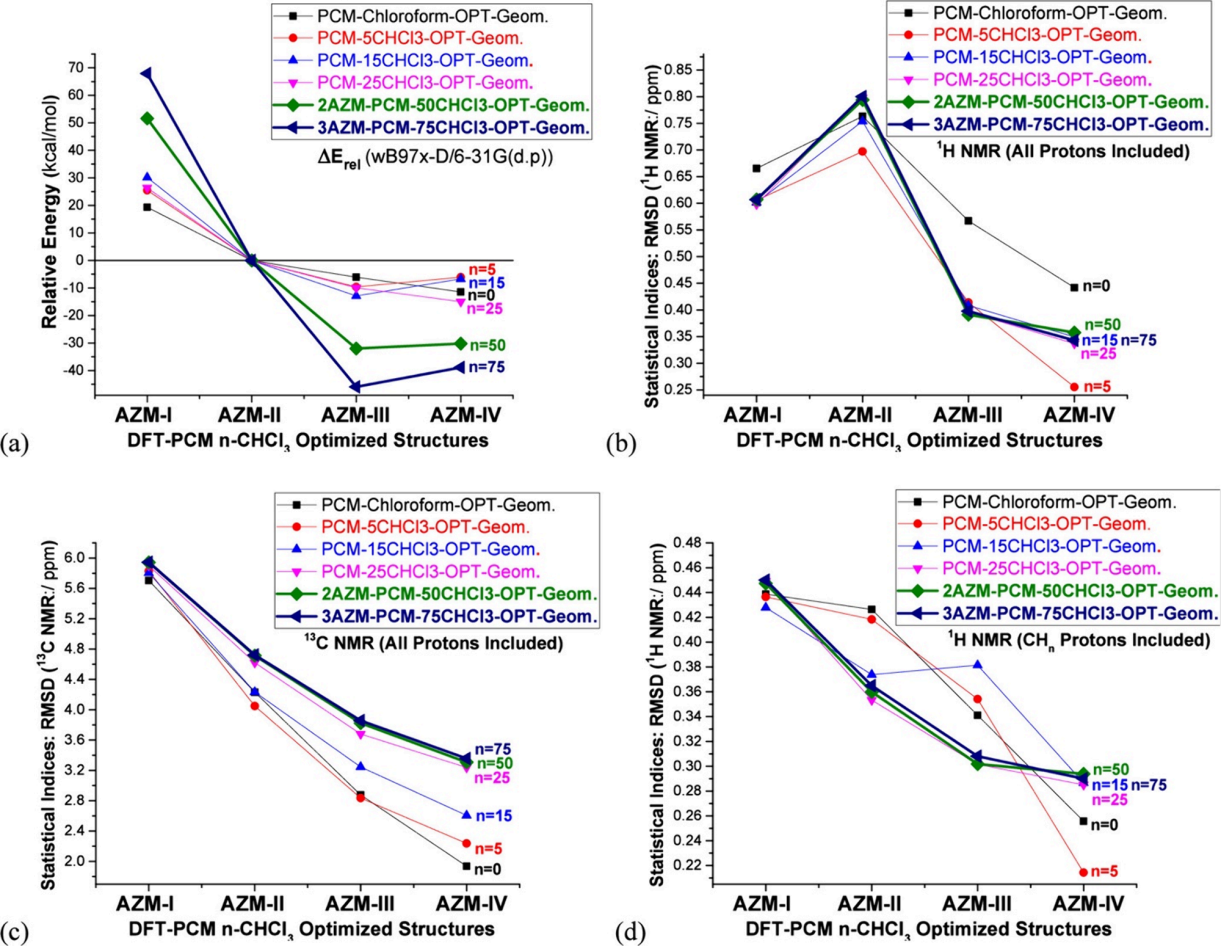
Relative electronic energies (Δ*E*_*rel*_) and RMSD for four conformers of azithromycin (AZM-I to AZM-IV), optimized using PCM with chloroform and various numbers of explicit CHCl_3_ molecules (*n* = 0–75). (a) Relative energies calculated at the ωB97x-D/6-31G­(d,p)-PCM level. (b) RMSD values for calculated vs experimental ^1^H NMR shifts (all protons included). (c) RMSD for ^13^C NMR shifts. (d) RMSD for ^1^H NMR considering only CH_n_ protons. Explicit solvation effects increasingly stabilize AZM-III and AZM-IV, with ^1^H NMR profiles showing enhanced agreement with experiment upon inclusion of up to 75 solvent molecules. While relative energies are strongly impacted by solvation, NMR RMSD values remain robust across solvation models, underscoring their reliability for conformer assignment in solution. Reproduced with permission from reference 292. Copyright (2025) American Chemical Society.

Numerous recent studies have emphasized the role of solvent modeling in improving the accuracy of computed NMR chemical shifts across a wide range of molecular systems. In one study, Cseri and co-workers^293^ conducted an experimental and computational investigation of ^1^H and ^13^C shifts in green solvents, acids, and organocatalysts, using the IEFPCM model in DMSO. While ^13^C shifts were reasonably captured, large errors were observed for ^1^H nuclei in protic and polar species, where continuum models failed to describe localized solute–solvent hydrogen bonding. These results highlighted the limitations of implicit solvation and pointed to the need for hybrid schemes. Live and colleagues^284^ examined ^15^N shifts in gramicidin S and found both local and long-range solvent influences, with protic solvents inducing downfield shifts via H-bonding and disruption of intramolecular H-bond networks. Temperature dependent experiments further revealed solvent modulated couplings across H-bonded amide chains, illustrating solvent’s role as both a direct interaction partner and a remote electrostatic modulator. In another systematic effort, Cohen et al.^294^ developed the DELTA50 benchmark data set and compared PCM, CPCM, and SMD models for ^1^H and ^13^C shift predictions in drug-like molecules. The SMD model showed improved performance, especially for functional groups involved in hydrogen bonding, emphasizing the importance of solvent model selection. Gao et al.^295^ evaluated ^11^B shift predictions in boron-containing species solvated in THF and showed that including solvent via SMD or CPCM significantly reduced RMSD, even in geometrically constrained systems. De Andrade’s group^296^ focused on ^99^Tc shielding in metal complexes and demonstrated that solvation using the COSMO model was necessary to reproduce deshielding trends in polar media, revealing the sensitivity of metal centered shifts to dielectric screening. *Ab initio* MD with explicit DMSO solvation was applied to Pt­(III) dinuclear complexes by Batista et al.,^297^ revealing that variations in Pt–N/O bond distances influenced ^195^Pt shifts effects that were not reproduced by implicit solvation models. Local orbital analysis further revealed that solvent polarization alters virtual orbital energies, driving deshielding. A fully explicit QM/MM protocol using COBRAMM^298^ was employed by Calcagno and co-workers to compute ^31^P shifts in aqueous environments. Their approach sampling hundreds of MD snapshots captured polarization and solvent fluctuation effects more accurately than cluster based or implicit treatments, advocating for explicit embedding in polar phosphorus systems. These studies show that accurate solvent modeling, whether using improved implicit models or fully explicit QM/MM frameworks, is essential for reliable and transferable NMR shift predictions.

The development of robust computational workflows for NMR chemical shift prediction has significantly advanced structural determination in chemistry, particularly for complex organic molecules. Willoughby et al.^299,300^ introduced a protocol designed to compute ^1^H and ^13^C NMR chemical shifts for structural validation, emphasizing accessibility for bench chemists with limited computational expertise. The workflow involves generating conformers using molecular mechanics software like MacroModel,^301,302^ followed by geometry optimization and frequency calculations via DFT with the Gaussian software.^303^ NMR shielding tensors are computed for each conformer, and Boltzmann-weighted chemical shifts are derived for comparison with experimental data, as outlined in Figure 3. This approach addresses constitutional and configurational ambiguities, enabling accurate structural assignments for natural products and synthetic compounds. The modular design allows adaptation to complex systems, despite challenges like identifying numerous conformers, extended computational times, and handling multiple candidate structures. By integrating computational rigor with practical usability, the protocol bridges experimental and computational methods, enriching molecular characterization with accurate and reproducible insights.

**Figure 3:**
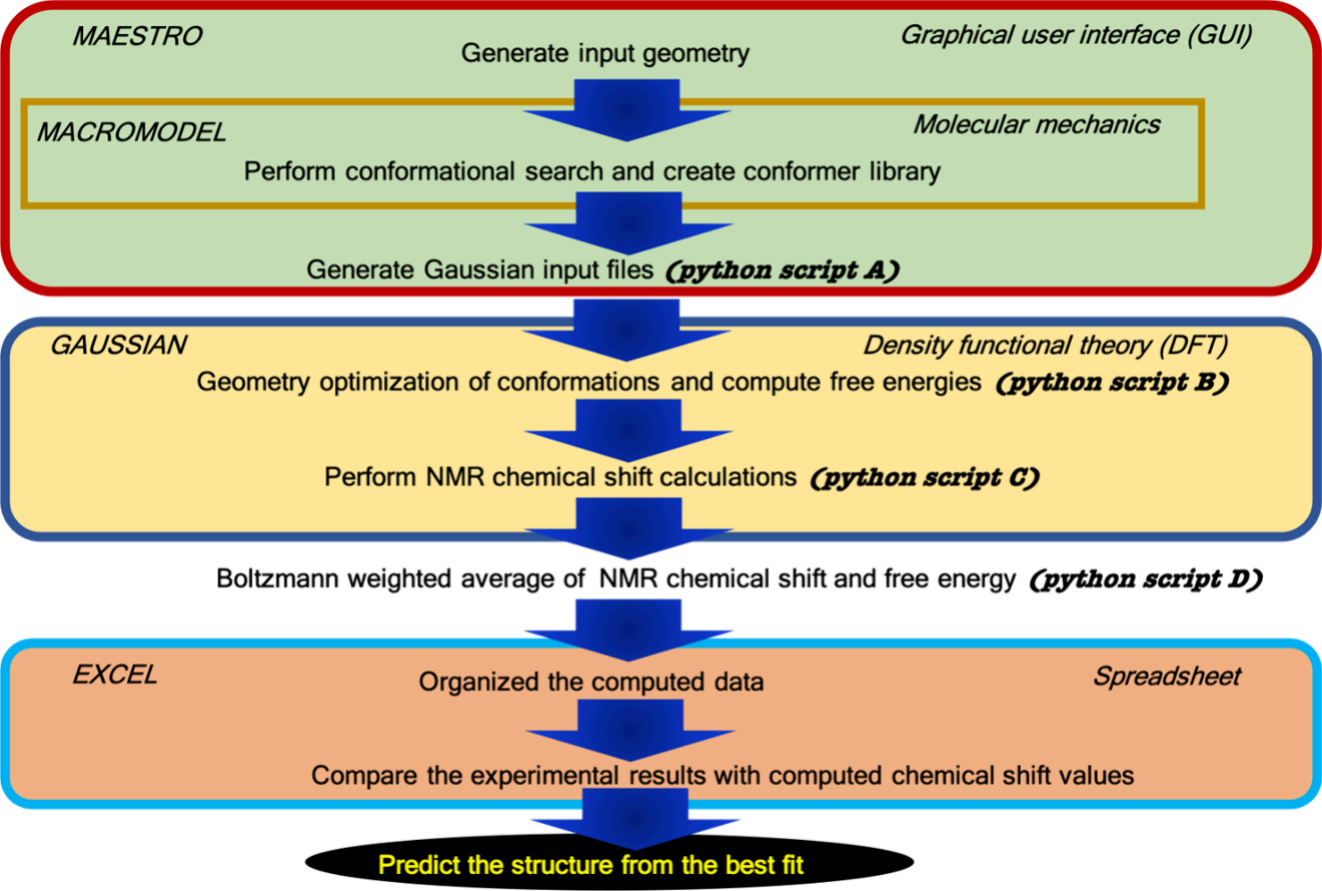
Schematic protocol for NMR chemical shift prediction as developed by Willoughby et al.^299^ The approach integrates molecular conformer generation, DFT-level NMR shift calculations, and statistical analyses to assign shifts to candidate structures. Reproduced with permission from reference 299. Copyright (2021) American Chemical Society.

Senanayake et al. introduced a workflow combining DFT computations with empirically derived error correction terms, providing a robust method for ^13^C NMR chemical shift prediction.^304^ The procedure (Figure 4) begins with candidate structures proposed based on experimental data, followed by conformational searches using MOE^305,306^ and the LowModeMD^307^ algorithm to identify potential energy minima. Conformers undergo geometry optimization in Gaussian.^303^ Solvation effects are accounted for using CPCM in solvents such as DMSO, chloroform, and methanol.^279,287^ ^13^C shielding constants are computed via the GIAO method, converted to chemical shifts relative to TMS or scaled with linear regression. Employing B3LYP/cc-pVDZ, the method balances accuracy and computational cost, achieving high reliability with systematic error corrections for functional groups. The workflow accelerates structure elucidation, particularly for pharmaceutically relevant compounds, by integrating computational predictions with experimental data to refine molecular characterizations. Its accessibility, combined with user-friendly tools, helps to bridge the gap between experimental and computational approaches.

**Figure 4:**
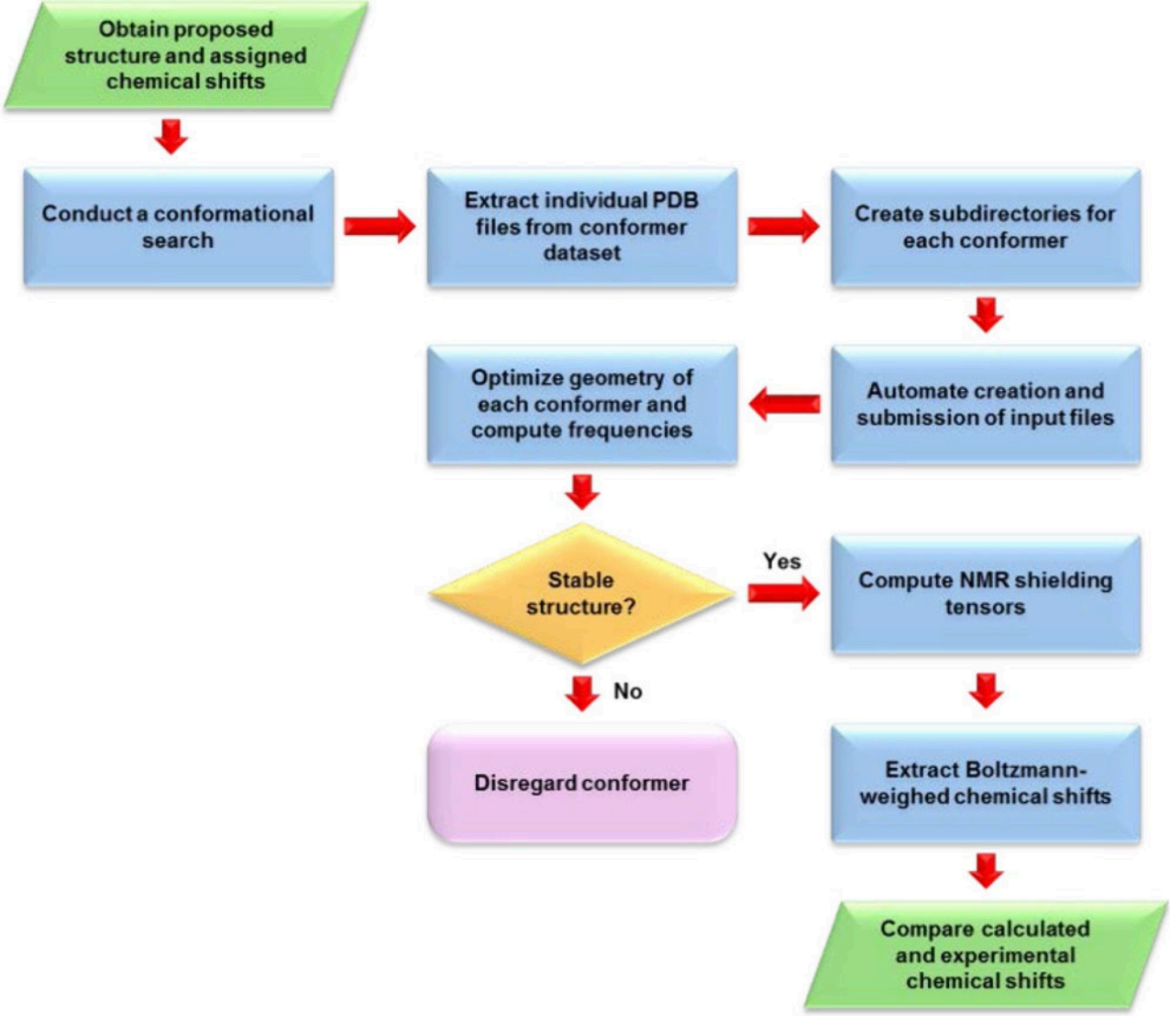
Workflow for computing NMR chemical shifts as described by Senanayake et al.^304^ The protocol involves conformer generation, geometry optimization, vibrational analysis, and DFT-based shielding tensor calculations, followed by Boltzmann weighting and comparison to experimental data. Reproduced with permission from reference 304. Copyright (2017) American Chemical Society.

Several automated approaches and workflows for predicting NMR-active nuclei chemical shifts have been developed to assist nonexperts in computational NMR, relying on high-level QM methods to produce accurate results. However, their computational cost limits applicability to large data sets, and complex molecules, such as metabolites with numerous rotatable bonds, which challenges existing workflows due to extensive conformational spaces.^308^ Current methods often lack validation for such cases, highlighting the need for a high-throughput, automated workflow capable of accurate predictions across diverse structures. Developing such methodologies would enable comprehensive NMR data libraries for complex molecules, advancing structural elucidation and large-scale metabolomics studies.

Merz et al.^97^ introduced an efficient computational workflow for predicting NMR chemical shifts of medium-sized organic molecules, including metabolites, with reduced computational costs and high accuracy. The workflow, depicted in Figure 5, combines force field, machine learning, and quantum mechanical methods, employing steps such as conformation generation, ML-based filtering using the ASE-ANI model,^309,310^ clustering,^311^ DFT-based optimization, and chemical shift calculation.^303,312,313^ This modular protocol, executed through a series of Python scripts, supports user-defined combinations of DFT theory, solvent models, and NMR-active nuclei. For high-throughput applications, the workflow can be fully automated using managers like Snakemake^314,315^ or the Galaxy web server,^316^ enabling seamless data processing and large-scale QM data set generation. By addressing computational efficiency and accuracy, the workflow provides a robust foundation for advancements in structural assignment and computational NMR applications.

**Figure 5:**
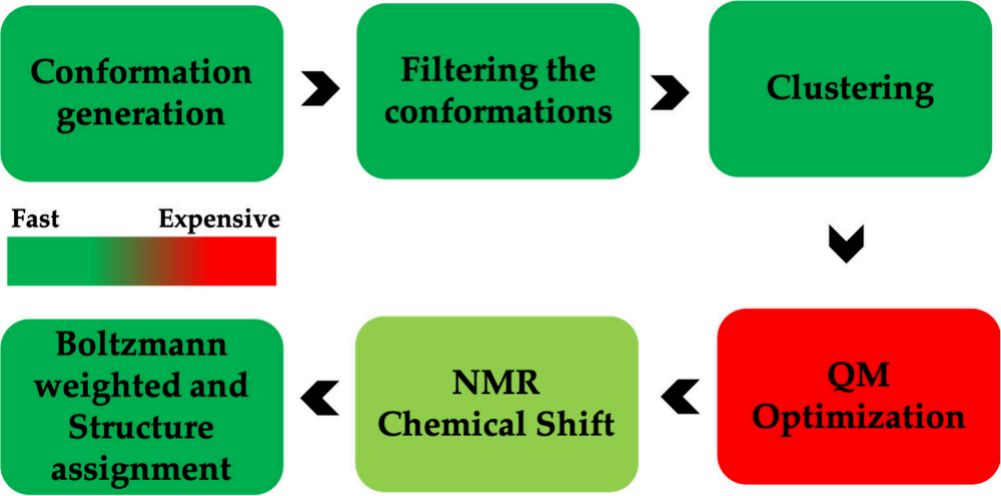
Schematic workflow for Boltzmann-weighted NMR chemical shift prediction as outlined by Merz et al.^97^ The process includes conformer generation, filtering, clustering, quantum mechanical optimization, and NMR shift calculations, followed by Boltzmann-weighted structure assignment. Reproduced with permission from reference 97. Copyright (2020) American Chemical Society.

While DFT-based calculations provide valuable insights into the dependence of chemical shifts on local geometry, empirical and semiempirical approaches have been more successful in modeling local backbone structures.^317^ Hunter et al. highlighted semiempirical methods for chemical shift evaluation,^318^ while Merz et al. advanced computational NMR through semiempirical and QM/MM methods to address challenges in large biological systems, including proteins and protein–ligand complexes.^109,319^ Semiempirical methods, using AM1 parameters, offer computationally efficient alternatives to *ab initio* and DFT calculations, enabling the modeling of macromolecules with acceptable accuracy and reduced costs. Merz et al. further enhanced NMR predictions with QM/MM approaches, partitioning systems into quantum and molecular mechanical regions, as demonstrated in the FKBP-GPI protein–ligand complex, where computed shifts aligned closely with experimental data. Their Automated Fragmentation QM/MM (AF-QM/MM)^111^ method refined shielding constant calculations, emphasizing conformational flexibility’s role in accuracy.^320^ Additionally, the HECSP model and NMRScore_P scoring function,^110^ developed to predict ligand-induced protein chemical shift perturbations and refine protein–ligand structures, demonstrated strong correlations with experimental data. These methodologies, depicted in Figure 6, underscore their usefulness in computational NMR, providing a robust framework for structural insights and therapeutic discovery advancements.

**Figure 6:**
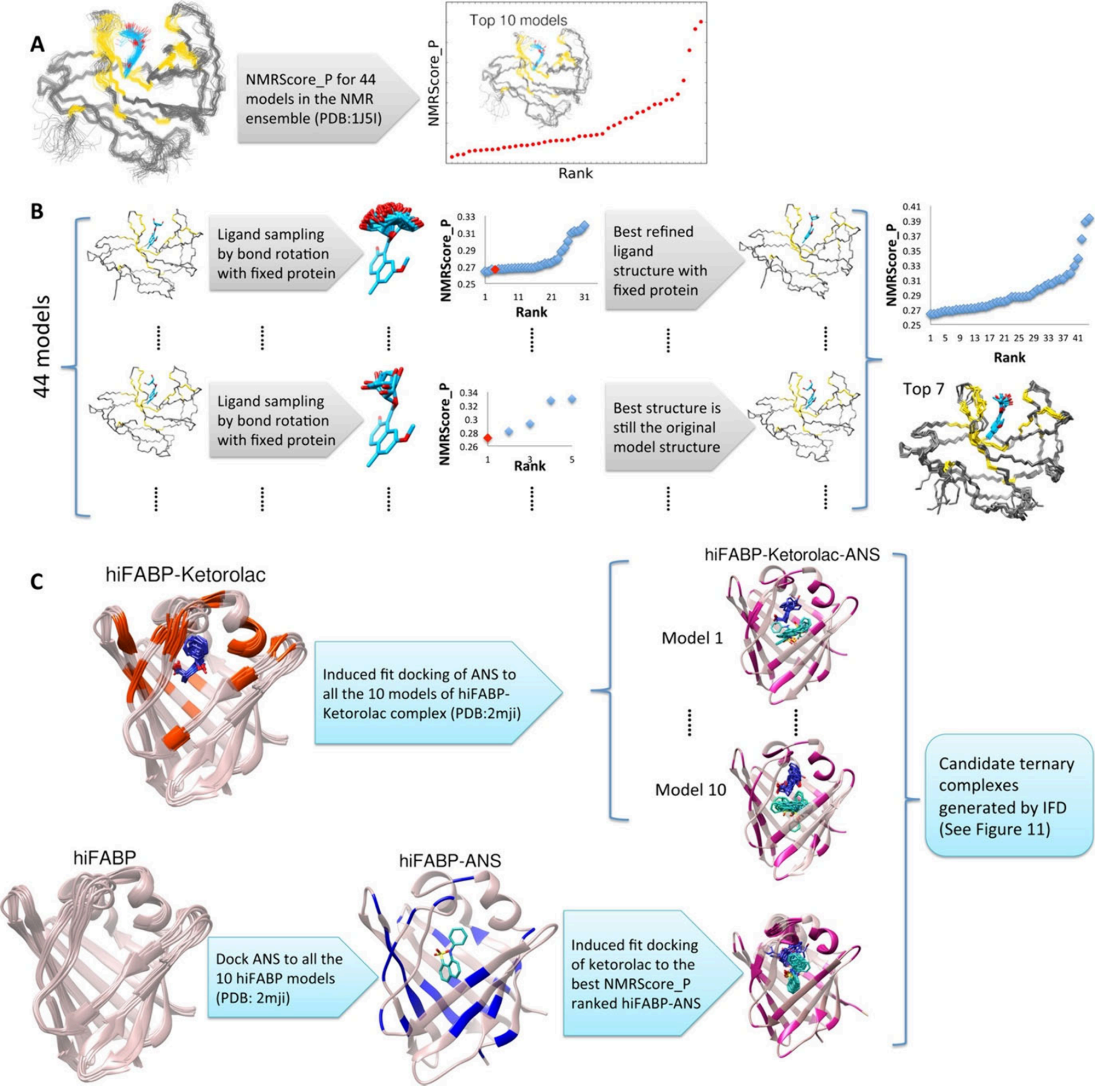
Merz et al.’s workflow for NMR chemical shift computation using NMRScore_P.^110^ (A) Workflow for ranking structures within the NMR ensemble. (B) Workflow for ligand structure refinement. (C) Workflow for structure determination. Reproduced with permission from reference 110. Copyright (2017) American Chemical Society.

While DFT remains the most widely used approach in NMR computation due to its favorable balance between accuracy and efficiency, its limitations become evident in systems with strong correlation, delocalized electrons, heavy atoms, or subtle vibrational and solvent effects. In such cases, DFT often fails to capture the necessary electron correlation and magnetic response properties with sufficient precision. To overcome these challenges, a range of post-DFT methods including MP2,^321,322^ CCSD/CCSD­(T),^323^ DLPNO–CCSD,^223,324,325^ SOPPA,^228^ and CASSCF/RASSCF^326^ have been employed to achieve higher accuracy and deeper physical insight. The following discussion highlights benchmark studies that demonstrate the advantages, limitations, and computational trade-offs of these high-level methods in NMR applications.

High-level electron correlation methods have played a critical role in advancing the predictive accuracy of NMR computation, particularly for nuclear spin–spin coupling constants (SSCCs) and shielding tensors. In a comprehensive benchmark by Gleeson et al.,^323^ vibrational corrections to SSCCs were evaluated across a chemically representative set of 21 small molecules including H_2_O, CH_4_, NH_3_, HCN, C_2_H_2_, CO, OHF, and OF_2_ using CCSD, CCSD­(T), and a range of cost-effective alternatives such as SOPPA, and SOPPA­(CCSD). Vibrational averaging was incorporated using second-order vibrational perturbation theory, and CCSD-based SSCCs combined with CCSD­(T) geometries and cubic force fields (denoted CCSD//CCSD­(T)) yielded the closest agreement with experiment (MAD ≈ 1.5 Hz). However, this high level of accuracy came with steep computational costs, restricting full CCSD­(T) treatment to molecules containing fewer than five heavy atoms due to the extensive demands of force and property derivative calculations. To mitigate this, mixed protocols were tested wherein high-level equilibrium values from CCSD were paired with vibrational corrections from B3LYP, yielding comparably accurate results (MAD = 1.9 Hz) at a fraction of the cost. Yet, SOPPA-based protocols such as SOPPA//MP2 and SOPPA­(CCSD)//MP2, while computationally more feasible for larger systems, introduced triplet instabilities and inaccuracies in geometrical configurations that diminished the benefit of including vibrational corrections. Similarly, in the studies by Faber and Sauer,^327^ as well as Jessen and Sauer,^328^ CCSD and SOPPA­(CCSD) were applied to predict ^19^F SSCCs across a diverse set of fluorinated systems, including CF_4_, CH_3_F, CHF_3_, C_2_F_6_, CH_2_CHF, CH_2_CF_2_, and fluorinated heterocycles and fluoroalkenes. These benchmark studies highlighted the importance of high-level correlation methods and vibrational corrections for achieving quantitative agreement with experimental ^19^F–^19^F coupling constants. The full CCSD approach, executed with large aug-cc-pCVQZ basis sets, achieved near-experimental accuracy (MAD ≈ 1.5–2 Hz), but remained computationally prohibitive for molecules with more than 10 heavy atoms. To balance fidelity and feasibility, SOPPA-level spin–spin coupling constants were combined with CCSD optimized geometries and CCSD-derived vibrational corrections in composite SOPPA//CCSD + CCSD protocols.^327,328^ This hybrid strategy preserved predictive accuracy while mitigating the computational expense of full CCSD treatments. However, the known basis set sensitivity of SOPPA and its inconsistent performance for π-conjugated and unsaturated systems underscore its limitations for routine high-accuracy applications.^329^ These studies collectively underscored that while coupled-cluster methods are ideal for benchmark level predictions, their practical scope is best extended through judicious method combinations tailored to system size and chemical complexity.

Efforts to improve computational efficiency without sacrificing accuracy have also been demonstrated through local correlation approaches. Among these, the domain-based local pair natural orbital (DLPNO) methods developed by Neese and co-workers have shown considerable promise for spin–spin coupling constant (SSCC) calculations in small to medium-sized molecules. Preliminary applications to halogenated and polar systems suggest encouraging agreement with canonical CCSD results at a fraction of the computational cost.^330–332^ The key advantage of DLPNO–CCSD lies in its reduced scaling, enabling systems of ∼ 30 atoms to be treated on standard computational infrastructure. Nonetheless, limitations were noted in delocalized systems such as benzene, where the localization of electron correlation induced marginal overlocalization errors. Complementing these coupled-cluster strategies, Da Silva et al. and Dittmer et al. demonstrated the utility of MP2 in modeling NMR parameters across both solution-phase and solid-state systems. Da Silva et al.^333^ studied ^1^H shifts for nitrogen-containing molecules, valerolactam, formyl anilide, *N*-methylbenzylamine, phenanthridone in CDCl_3_ using MP2/6-31G­(d,p) with GIAO and PCM, revealing substantial errors for N–H shifts (up to −1.8 ppm) when explicit solvent molecules were not included. Upon inclusion of three to four solvent molecules, N–H agreement improved, albeit at the cost of degraded C–H accuracy. Dittmer et al.^325^ applied MP2 within an embedded cluster model to predict ^13^C and ^15^N shieldings in urea and formamide crystals, showing improved accuracy over periodic DFT but limited by system size and model complexity. For multireference systems, methods such as CASSCF and RASSCF combined with GIAOs have been applied to compute ^15^N shieldings with improved accuracy. Nottoli et al.^334^ implemented a Cholesky-decomposed CASSCF-GIAO scheme capable of treating systems with ∼ 1,300 basis functions. RASSCF offers a scalable alternative by partitioning active spaces (RAS1/2/3) and is suitable for larger systems.^335^ Gauge-origin independence remains critical, typically addressed via GIAOs.^163^ These approaches help correct SCF-level errors in ^15^N shifts, which can exceed tens of ppm in strongly correlated systems. Meanwhile, Gendron et al.^326,336^ used CASSCF with spin–orbit CI to interpret ^1^H and ^13^C shifts in 5f actinide cyclopentadienyl complexes, resolving spin–orbit and magnetic anisotropy effects inaccessible to DFT. Despite offering physically grounded insight, both RASSCF and CASSCF approaches remained computationally expensive and highly sensitive to active space definition, limiting their routine use to systems with ≤ 30 correlated orbitals. These studies suggest that while high-level correlated methods are required for reliable NMR modeling, their broader application must be guided by a balance between electronic accuracy and computational cost.

The application of relativistic corrections has become essential in the accurate calculation of NMR parameters, including chemical shifts and J-couplings, particularly for molecules containing heavy elements or those influenced by heavy-atom neighbors. Pyykko eloquently framed the broader significance of these corrections, emphasizing that relativistic effects are “more common than you thought,” and highlighting their role in a wide range of chemical phenomena, from the color of gold to the covalency trends in f-block complexes, as well as systematic errors in computed NMR parameters if neglected.^196^ Comprehensive reviews and studies by Autschbach and co-workers^170^ have demonstrated that relativistic quantum chemistry methods, including two- and four-component approaches, are now routinely applied to evaluate nuclear magnetic shielding and indirect spin–spin coupling tensors, with DFT providing a practical framework for large and heavy-element systems. Repisky et al.^337^ have detailed the implementation and application of these methods, showing that both scalar and spin–orbit effects can be variationally included to achieve accurate NMR parameters for molecules with heavy atoms. Practical improvements in calculated chemical shifts, often by hundreds of ppm, have been documented for transition metal and main-group compounds when quasirelativistic corrections are included, as shown in studies on molybdenum and tungsten complexes.^338^ Reviews by Autschbach and others have also cataloged heavy-neighbor effects, where even lighter atoms in proximity to heavy elements exhibit significant relativistic influences on their NMR shifts.^172,193,337–339^ The HALA (heavy-atom effect on light-atom) and HAHA (heavy-atom effect on heavy-atom) mechanisms, as discussed by M. Kaupp, have been used to interpret changes in shielding tensors due to heavy-atom environments.^340^ Applications have spanned main-group, transition-metal, and actinide compounds, where relativistic contributions are consistently found to be significant. Early theoretical work, such as that of Ramsey and subsequent developments, established the foundation for incorporating relativistic corrections into magnetic resonance parameter theory.^239,341^ These applications underscore the necessity of relativistic treatments for reliable NMR predictions in modern computational chemistry.

In summary, QM methods have significantly advanced computational NMR, offering precise insights into structural biology, metabolomics, and materials science. High-accuracy approaches, such as CCSD­(T) and DFT, have enabled reliable predictions of chemical shifts and J-couplings, aiding stereochemical resolution, structural prediction, and protein–ligand interaction analysis. However, challenges persist, including high computational costs, limited scalability for large biomolecules, and dependencies on functionals, basis sets, and approximations. Limitations in modeling solvent effects and conformational flexibility further complicate applications. Machine learning models trained on extensive NMR data sets are emerging as a scalable solution, enabling rapid predictions of NMR parameters. The integration of ML techniques represents a transformative step, bridging the precision of quantum chemistry with the scalability required for real-world systems, thus expanding the scope of computational NMR.

## 3 Machine Learning Approaches in Computational NMR

### 3.1 Role of Machine Learning

Empirical approaches often neglect stereochemical and conformational effects, limiting their predictive capacity, while quantum chemical methods are computationally prohibitive for large or complex systems due to their resource-intensive nature. To overcome these limitations, machine learning has emerged, offering scalable and efficient solutions for NMR parameter prediction and spectral interpretation.^117,342^ By leveraging large data sets, ML algorithms identify patterns and correlations, enabling rapid predictions without relying on first-principles calculations. This data-driven approach has demonstrated remarkable success across diverse applications, including small-molecule characterization,^343,344^ metabolomics,^345–348^ and structural biology,^349–351^ while also addressing the computational challenges posed by traditional methods. Furthermore, ML methods have been applied to correlate experimental and calculated NMR data, facilitating structural elucidation and expanding the accessibility of spectroscopic analysis.^352–354^ As the field continues to evolve, advances in areas such as spin–spin coupling constant prediction, paramagnetic system analysis, and the integration of ML with molecular dynamics simulations are driving new innovations in NMR. We highlight key contributions in this area, focusing on essential studies while acknowledging the breadth of important work in the field. The selected articles provide an overview of the field and highlight future prospects for this rapidly evolving research area.

### 3.2 Data Resources for ML-Driven NMR Applications

Databases like NMRShiftDB,^355,356^ the Natural Products Magnetic Resonance Database (NP-MRD),^357^ the Biological Magnetic Resonance Data Bank (BMRB),^358,359^ the Cambridge Structural Database (CSD),^360,361^ and the CHESHIRE database^249,362,363^ are essential resources for advancing machine learning applications in NMR spectroscopy. NMRShiftDB, an open-access database, hosts around 400,000 experimental ^13^C chemical shifts, along with ^1^H data, covering natural products, synthetic compounds, and biomolecules. Its curated entries include chemical structures, assigned shifts, and metadata such as solvent conditions and reference standards, providing high-quality training data sets that enable ML models to achieve accurate chemical shift predictions, often rivaling quantum chemical methods. NP-MRD offers a vast repository of NMR data on over 281,859 natural products, including 5.5 million spectra, supporting metabolomics and other omics-based research fields, with enhanced spectral search tools facilitating prediction and analysis. BMRB specializes in biomolecular NMR data, providing detailed ^1^H, ^13^C, and ^15^N assignments for proteins, nucleic acids, and metabolites, aiding ML in tackling complex biological systems. The CHESHIRE database, a specialized resource for computed NMR scaling factors, provides ^13^C and ^1^H data alongside coupling constants, supporting the integration of chemical shifts into predictive workflows. CSD complements these resources by linking crystallographic and NMR data to refine structural predictions. The newly developed DFT8K data set comprises 8,000 DFT-optimized structures with 200,000 computed chemical shifts.^344,364^ Other specialized tools, such as GISSMO,^365,366^ optimize spin system matrices through experimental data alignment. These curated databases with comprehensive metadata provide diverse, contextualized data sets critical for training ML algorithms, fostering collaborative and open-science initiatives.

A distinctive advantage of NMR over other analytical platforms, such as LC-MS or chromatography, lies in its capacity to generate high-fidelity computational data grounded in quantum mechanics. Whereas most ML models in LC-MS must rely on large volumes of experimental data often constrained by noise, limited chemical diversity, or ambiguous annotations, NMR allows for the creation of large-scale, chemically diverse data sets from first-principles calculations.^132,367^ This stems from the fact that NMR observables, such as chemical shifts and scalar couplings, are directly linked to the underlying electronic structure of molecules and can be accurately computed using DFT, MP2, or CCSD methods. As a result, ML models can be trained on computed data in regimes where experimental data is sparse or unavailable. This strategy has been used effectively in models such as CASCADE^112^ and ML-J-DP4,^115^ which leverage computed chemical shift data sets to improve spectral prediction and structural assignment. However, computed data may omit important experimental nuances such as conformational averaging, solvent effects, or systematic instrument-related biases. Therefore, hybrid approaches-which combine the breadth and reproducibility of computed data with the accuracy and realism of experimental data sets are emerging as the most effective strategy for training robust, generalizable models. This hybrid paradigm positions NMR as a uniquely computable and scalable foundation for developing next-generation predictive tools.

### 3.3 Application and Challenges of ML models for NMR Computation

Applications of machine learning in computational NMR span a wide range of methodologies, including supervised learning,^368–370^ unsupervised learning,^371^ deep learning,^372–374^ and generative models,^345,375^ each addressing distinct challenges. Supervised methods, such as support vector machines (SVMs), random forests, and deep neural networks (DNNs), predict chemical shifts with high accuracy by leveraging structural descriptors like atomic charges and interatomic distances.^376–385^ Unsupervised learning techniques, including k-means clustering, hierarchical clustering, and Gaussian mixture models, assist in peak assignment, spectral clustering, and metabolite identification by uncovering hidden patterns in unlabeled data sets.^386–389^ Dimensionality reduction methods, such as principal component analysis (PCA) and t-SNE, reduce data complexity, facilitating the analysis of high-dimensional NMR spectra.^390^ Deep learning models, particularly convolutional neural networks (CNNs)^391,392^ and recurrent neural networks (RNNs),^116,393^ excel at processing spectral data and molecular representations (e.g., graphs or SMILES) to predict chemical shifts or classify peaks in noisy, overlapping regions. Generative models, such as variational autoencoders (VAEs) and generative adversarial networks (GANs), enable the generation of novel molecular conformations and synthetic NMR spectra, enhancing training data sets and bridging the gap between 2D and 3D structural data.^394^ Hybrid approaches combining QM with ML methods for chemical shift predictions help improve scalability and precision. Deep reinforcement learning (RL) has also been explored for automating structural elucidation tasks.^395,396^ Despite their advantages - speed, scalability, and robustness to noise - these methods face challenges such as the need for large, high-quality data sets and the interpretability of complex models. Explainable AI methods are increasingly addressing these issues, fostering confidence in ML-driven NMR applications.^397,398^

By utilizing the aforementioned data sets, Paton et al. recently developed a ML model, CASCADE^364^ (depicted in Figure 7), for NMR chemical shift predictions. The model builds upon three GNN architectures: DFTNN, ExpNN-dft, and ExpNN-ff. The DFTNN model was trained on the DFT8K data set, which includes chemical shifts computed at the mPW1PW91/6-311+G­(d,p)^399^ level and geometries optimized at the M06–2X/def2-TZVP level.^400^ To enhance accuracy, the authors employed transfer learning to retrain DFTNN against 5,000 experimental chemical shifts from the Exp5K data set, resulting in the ExpNN-dft model. However, the need for structure optimization in ExpNN-dft posed a computational bottleneck, which was addressed by ExpNN-ff, where force field-derived geometries (MMFF94) replaced QM-optimized structures. This optimization reduced the CPU time dramatically. Differences between GNN-predicted chemical shifts and DFT-calculated values yielded mean absolute errors (MAE) of 1.26 ppm for ^13^C and 0.16 ppm for ^1^H, with ExpNN-ff achieving comparable accuracy to DFT while being 5,000–10,000 times faster. Inspired by Roitberg et al., the authors incorporated high-quality CCSD­(T) values and demonstrated that molecular geometries from inexpensive calculations could maintain predictive accuracy.^309,310,401,402^ CASCADE effectively predicts chemical shifts for large, flexible molecules and addresses challenges in structure elucidation, data reassignment, and regioselectivity predictions.

**Figure 7:**
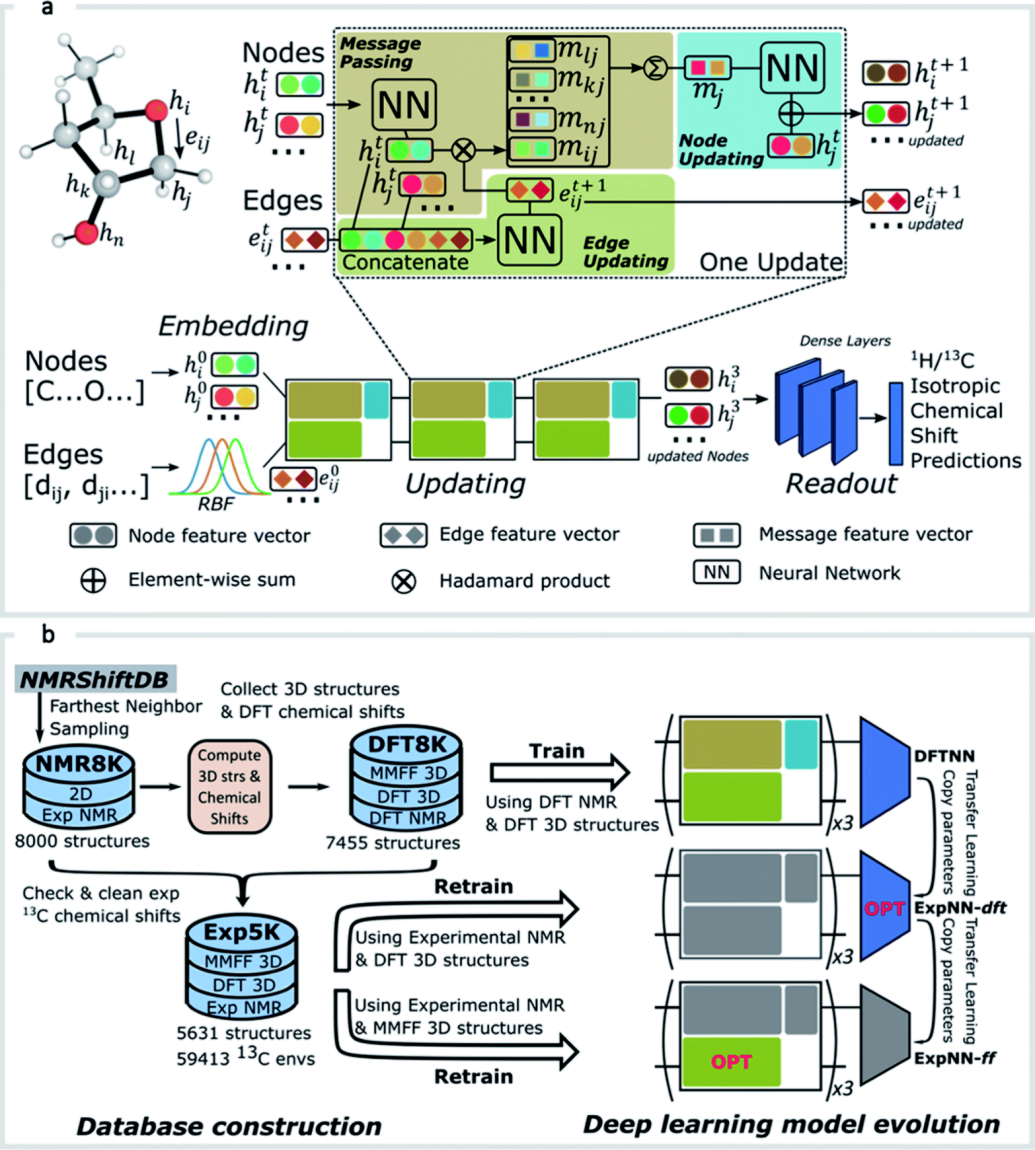
(a) Schematic representation of the GNN architecture used in CASCADE,^112^ illustrating the workflow from molecular input to model training and chemical shift prediction. Molecules are encoded based on atom types and interatomic distances, with iterative message-passing used to generate atomic representations for final predictions. (b) Workflow of data set generation and model development: NMR8K contains unchecked experimental chemical shifts and 2D structures; DFT8K augments this with DFT-optimized 3D structures and calculated shifts; Exp5K includes cleaned experimental shifts. Models trained on these data sets enable rapid and accurate ^1^H and ^13^C shift predictions. Schematic representation of the machine learning model used in CASCADE, illustrating the workflow from data preprocessing to model training and prediction. Reproduced with permission .^112^ *Copyright (2021) Royal Society of Chemistry*.

Building on the design strategy established in CASCADE, Yang and co-workers^403^ proposed a graph neural network architecture that predicts NMR chemical shifts directly from molecular graphs using a message-passing framework, eliminating the need for expert-designed features such as dihedral angles or electronegativity-based descriptors. The model processes molecular fragments where each atom is connected to both covalent and spatially adjacent neighbors through feature-rich edges containing information on bond type and interatomic distance. The architecture comprises edge embeddings, message passing layers with residual connections, and a final readout network that outputs chemical shifts for carbon, nitrogen, and hydrogen atoms. The overall network structure is illustrated in Figure 8. Unlike earlier models that rely on precomputed torsional data or sequence-derived features, this model is trained end-to-end using only atom types and structural graphs, making it applicable to both macromolecules and small organic species. The authors trained the model using three data sets that include 2405 protein structures from the RefDB database,^404^ curated examples from a previously established data set, and 369 metabolites from the Human Metabolome Database.^405^ The combined training regime produced over 393,000 protein fragments and several thousand labeled metabolite shifts, allowing the model to generalize across chemically diverse systems.

**Figure 8:**
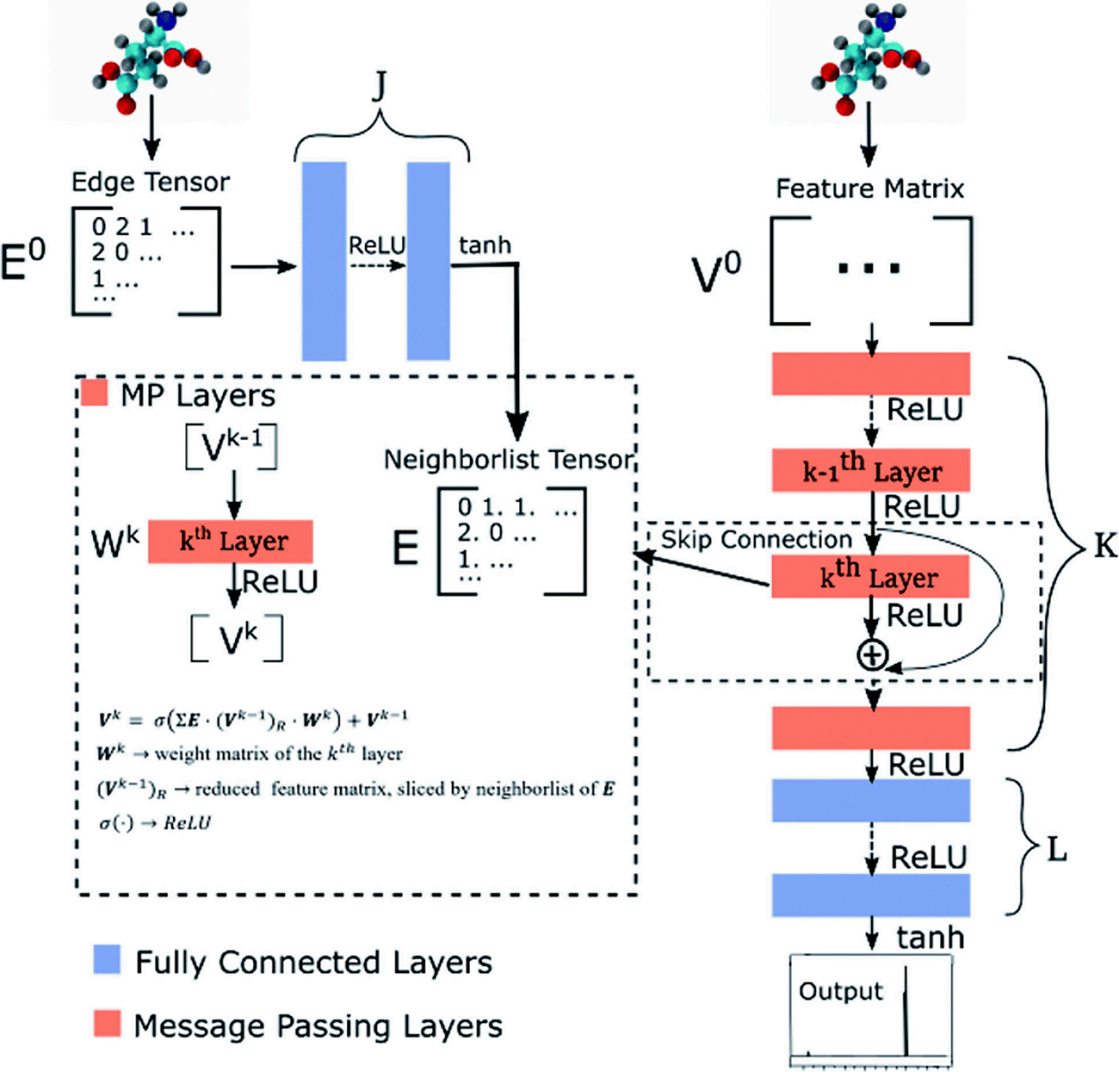
Graph neural network architecture for NMR chemical shift prediction. The model takes molecular graphs as input, where E^0^ encodes edge features such as inverse interatomic distances and bond types, and V^0^ represents the atomic feature matrix based on element types. These inputs are processed through K -MP layers with residual connections, using neighbor list tensors E to propagate local structural information. The resulting atomic representations are passed through L -FC layers, culminating in chemical shift predictions adjusted for element-specific effects. Red and blue blocks represent MP and FC layers, respectively. Reproduced with permission from reference 403. Copyright (2021) Royal Society of Chemistry.

Performance benchmarks show that the model achieves root mean squared deviations of 0.29 ppm for *H*_*α*_ atoms and correlation coefficients approaching 0.88, comparable to or better than existing models under identical input constraints. Furthermore, the model is capable of evaluating one million shifts in approximately five seconds using a single V100 GPU, demonstrating its suitability for high-throughput applications. Importantly, the model captures physically meaningful effects without being explicitly trained on them. It reproduces the downfield shift of *H*_*α*_ protons in β sheet structures and predicts chemical shift changes upon breaking a salt bridge between charged residues, aligning well with experimental values reported by White et al.^406^ These capabilities make the model well suited for integration into simulation-based structure inference workflows such as metadynamics and ensemble refinement. However, several challenges remain. The authors observed that accuracy decreases slightly when extending the model to multitype atom prediction due to increased data heterogeneity. The model also depends heavily on data quality, as more carefully curated subsets produce superior training curves compared to larger but noisier sets. Additionally, while distance features improved performance marginally, the impact of different graph featurization strategies remains underexplored. Future research will benefit from the development of larger and cleaner data sets, optimization of graph construction protocols, and the inclusion of richer edge features to better encode nonbonded interactions.

A new ML model, ML-J-DP4,^115^ developed by Sarotti et al., significantly accelerates structural elucidation with impressive accuracy and efficiency. ML-J-DP4 builds on the J-DP4 framework, a modification of DP4 that incorporates scalar ^3^J_HH_ couplings to constrain conformational sampling (iJ-DP4) and provides relevant stereochemical insights via a Bayesian probability term (dJ-DP4). Striking an optimal balance between accuracy and computational cost, J-DP4 has been recognized as one of the most effective methods for resolving highly complex molecular structures. ML-J-DP4 further enhances this approach by integrating Karplus-type J-coupling calculations with isotropic shielding predictions at the HF/STO-3G level, refined through Δ-ML techniques. As illustrated in Figure 9, the workflow involves selecting 17,000 molecules (T17k set) for training and 10,000 molecules (V10k set) for validation from an initial data set of 170,000 structures, achieving mean absolute errors (MAE) of 1.21 ppm for 13C and 0.14 ppm for ^1^H, with RMS values of 1.63 and 0.19 ppm, respectively. This fully automated workflow, combining Bayesian inference, adaptive learning, and molecular environment refinement, solves complex structures in minutes using standard computational resources. The method’s speed, accuracy, and efficiency make it an important development in applying machine learning to NMR.

**Figure 9:**
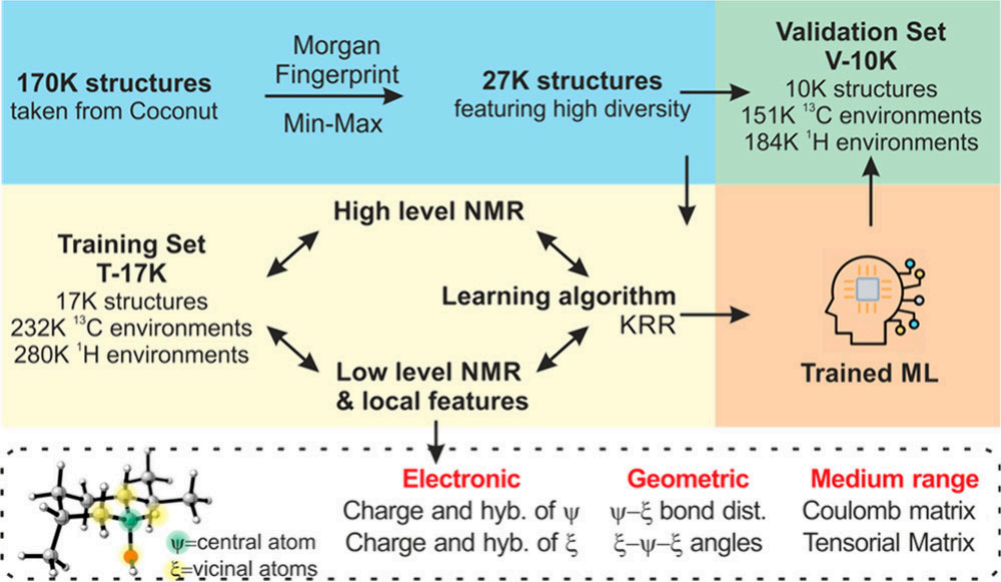
Schematic overview of the ML-J-DP4 method,^115^ illustrating a hybrid workflow that combines fast Karplus-type scalar coupling (^3^J) approximations with ML-refined NMR chemical shift predictions at the HF/STO-3G level. The process accelerates structural elucidation within the J-DP4 formalism, achieving near-DFT accuracy at significantly reduced computational cost. Reproduced with permission from reference 115. Copyright (2022) American Chemical Society.

Building upon these developments, an improved statistical framework, DP4+, was introduced by Grimblat, Zanardi, and Sarotti^407^ to address key deficiencies of the original DP4 ^408^ method. In response to the well recognized limitations of DP4, particularly its reliance on scaled shift errors and limited accuracy for closely related isomers, DP4+ was formulated with refined error modeling, enhanced prediction accuracy, and chemically informed treatments of NMR data. This enhanced probability model incorporates two foundational improvements. First, it introduces unscaled chemical shift errors alongside traditional scaled ones to better capture environment-specific deviations that are otherwise masked by linear regression. Second, it employs more accurate levels of quantum chemical theory, specifically using B3LYP/6-31G­(d) geometries and computing shielding tensors at PCM/mPW1PW91/6-31+G­(d,p) level. Benchmarking across a challenging data set of 48 isomeric compounds, DP4+ demonstrated superior performance over traditional DP4, correctly identifying the right structure in cases where the original method failed or provided overconfident yet incorrect predictions. For example, the aldol stereoisomer later confirmed as the correct structure and the natural product cryptomoscatone D2 were both confidently misidentified by the original DP4 method, with confidence scores exceeding 90%. In contrast, DP4+ correctly reassigned their stereochemistry with greater than 99% certainty, underscoring its ability to minimize false positives and enhance structural reliability. This improvement is traced not only to better shielding tensor calculations but also to the treatment of sp^2^ and sp^3^ hybridized atoms with separate t-distribution models, resulting in sharper statistical resolution. The performance gain was further quantified using a scoring system that rewards confident assignments: DP4+ achieved scores up to 2.4 times higher than DP4, particularly when both proton and carbon data were integrated. These results collectively suggest that DP4+ provides a more trustworthy and chemically nuanced approach to structure validation using NMR.

One of the key unresolved challenges is the dependency of performance on the accuracy and consistency of the statistical parameters, which must be recalibrated for each new level of theory used in shielding calculations. Although the authors validated the robustness of their parameter set across both training and validation data sets, the complexity of deriving and updating these parameters remains a barrier to broader adoption. Another limitation involves conformational flexibility, which is only partially addressed via Boltzmann-weighted averaging, and may still compromise accuracy in dynamic or solvent-sensitive systems. Future research is expected to refine the parametrization process by leveraging machine learning for adaptive error modeling and to further dissect the respective roles of ^1^H and ^13^C data. Interestingly, DP4+ was shown to correct misassignments when either ^1^H and ^13^C data alone failed, reinforcing the value of dual-nucleus approaches. The authors also emphasize that while ^1^H data often exerts stronger discriminative power, this effect varies with the choice of functional and basis set, warranting further studies on nucleus-specific prediction strategies. Overall, DP4+ stands as a critical advancement in NMR-driven structure validation, offering both improved accuracy and a framework for future developments in probabilistic and chemically informed structure elucidation strategies.

The development of ML models for predicting NMR chemical shifts faces several challenges, including the scarcity of high-quality experimental NMR data, solvent effects, and the computational complexity of traditional methods. The limited availability of accurate experimental ^1^H NMR spectra, particularly for biologically relevant compounds, hampers the ability to create reliable predictive models. Additionally, solvent interactions, which significantly influence chemical shifts, are often neglected in many existing models, leading to inaccuracies when predictions are applied across different solvents. Traditional QM methods, while highly accurate, are time-consuming and not feasible for large-scale applications. In this context, PROSPRE (PROton Shift PREdictor)^344^ emerges as a notable advancement in the field, as it addresses these challenges by leveraging a carefully curated, solvent-aware data set and employs a GNN for efficient, accurate predictions. A flowchart illustrating the PROSPRE methodology is depicted in Figure 10. PROSPRE is capable of accurately predicting ^1^H chemical shifts (mean absolute error <0.10 ppm), while outperforming previous models, and has been widely applied to over 600,000 biologically relevant compounds. By incorporating solvent-specific corrections, PROSPRE ensures more accurate predictions for compounds in various solvents, particularly water, methanol, chloroform, and dimethyl sulfoxide, making it a useful tool for metabolomics, drug discovery, and natural product research.

**Figure 10:**
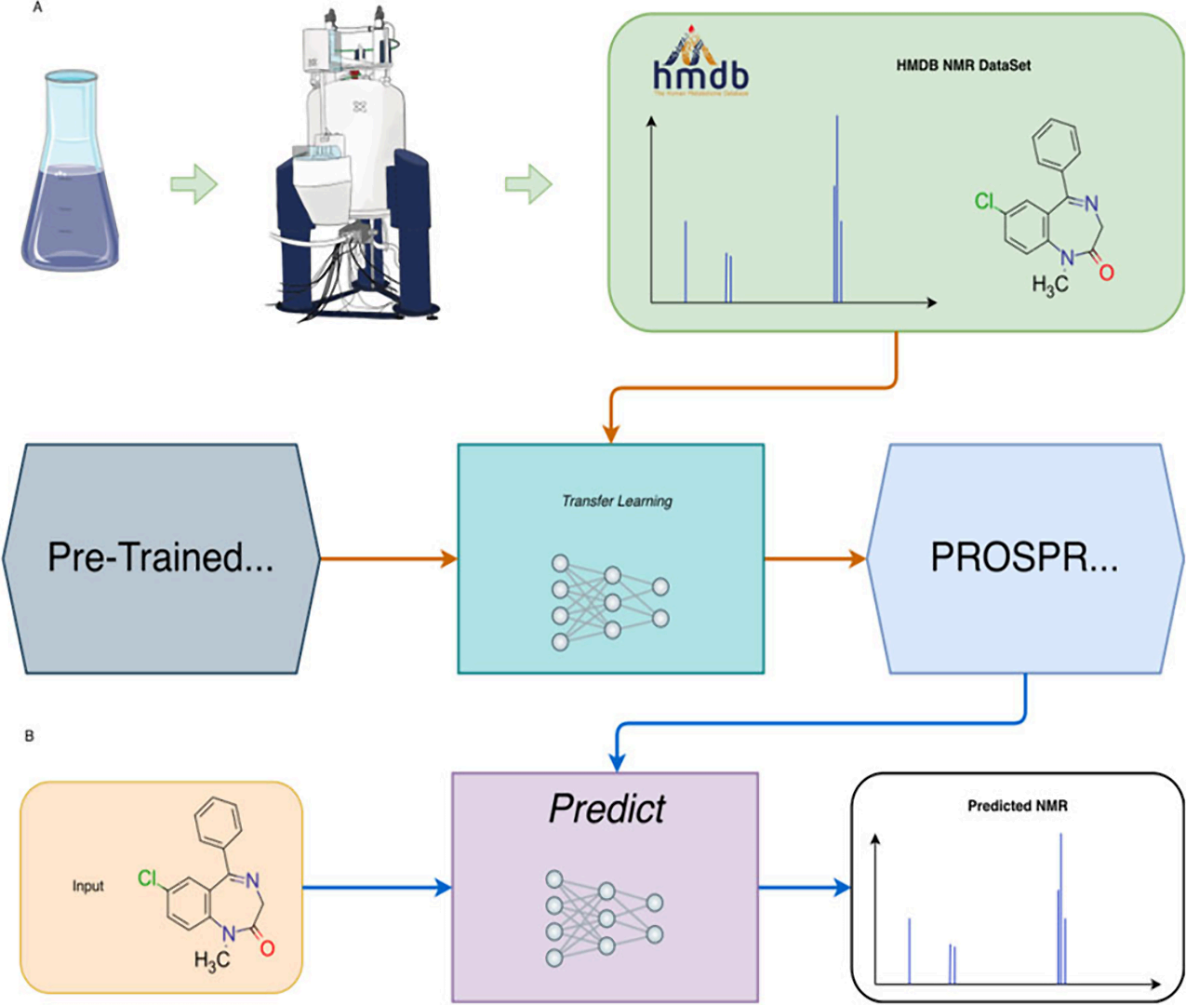
A schematic flowchart illustrating (A) the transfer learning methodology employed to train the PROSPRE GNN model, and (B) the workflow for predicting ^1^H chemical shifts from chemical structures using the PROSPRE model. Reproduced with permission from reference 344. Copyright (2024) by MDPI, Basel, Switzerland, under the Creative Commons Attribution (CC BY) license.

Recent advancements have significantly improved the accuracy of ^1^H NMR chemical shift predictions for small molecules by addressing limitations of existing experimental databases and incorporating solvent-aware GNN architectures.^112–114,344^ Extending these approaches to larger, more complex biomolecular systems such as proteins, however, introduces additional challenges, including the accurate representation of protein secondary and tertiary structures, solvent exposure, and conformational flexibility. The recently introduced EFG-CS model by Gu et al.^409^ addresses these challenges by integrating sequence-based protein structure prediction and chemical shift estimation into a unified computational pipeline. EFG-CS leverages protein structures predicted from amino acid sequences by the ESMFold algorithm, which uses deep-learning methods trained on extensive sequence data. Chemical shift predictions for backbone atoms are carried out by a transfer-learning-based ML model, utilizing alignment-derived structural and sequence information from reference databases. Additionally, side-chain atom chemical shifts are computed through a GNN-based model, incorporating spatial relationships between atoms within predicted protein structures. The overall computational pipeline employed by EFG-CS is depicted in Figure 11. In performance evaluations against the UCBShift test set^410^ of 200 nonredundant proteins, EFG-CS achieved root-mean-square errors (RMSEs) of 0.43 ppm for H, 0.27 ppm for Hα, 1.13 ppm for Cα, and 2.51 ppm for N atoms, using only ESMFold-predicted structures as input. Despite a modest performance drop relative to models using experimental structures, the results remain robust and comparable in accuracy, underscoring the model’s utility in scenarios where empirical structural data are unavailable. Notably, the prediction errors for *H*, *H*_*α*_, and *C*_*α*_ were tightly clustered around zero, whereas N atoms exhibited greater spread due to inherent challenges in modeling their diverse electronic environments. By eliminating the need for homology modeling or curated experimental input, the EFG-CS pipeline advances the accessibility and scalability of chemical shift prediction in data-limited or high-throughput settings.

**Figure 11:**
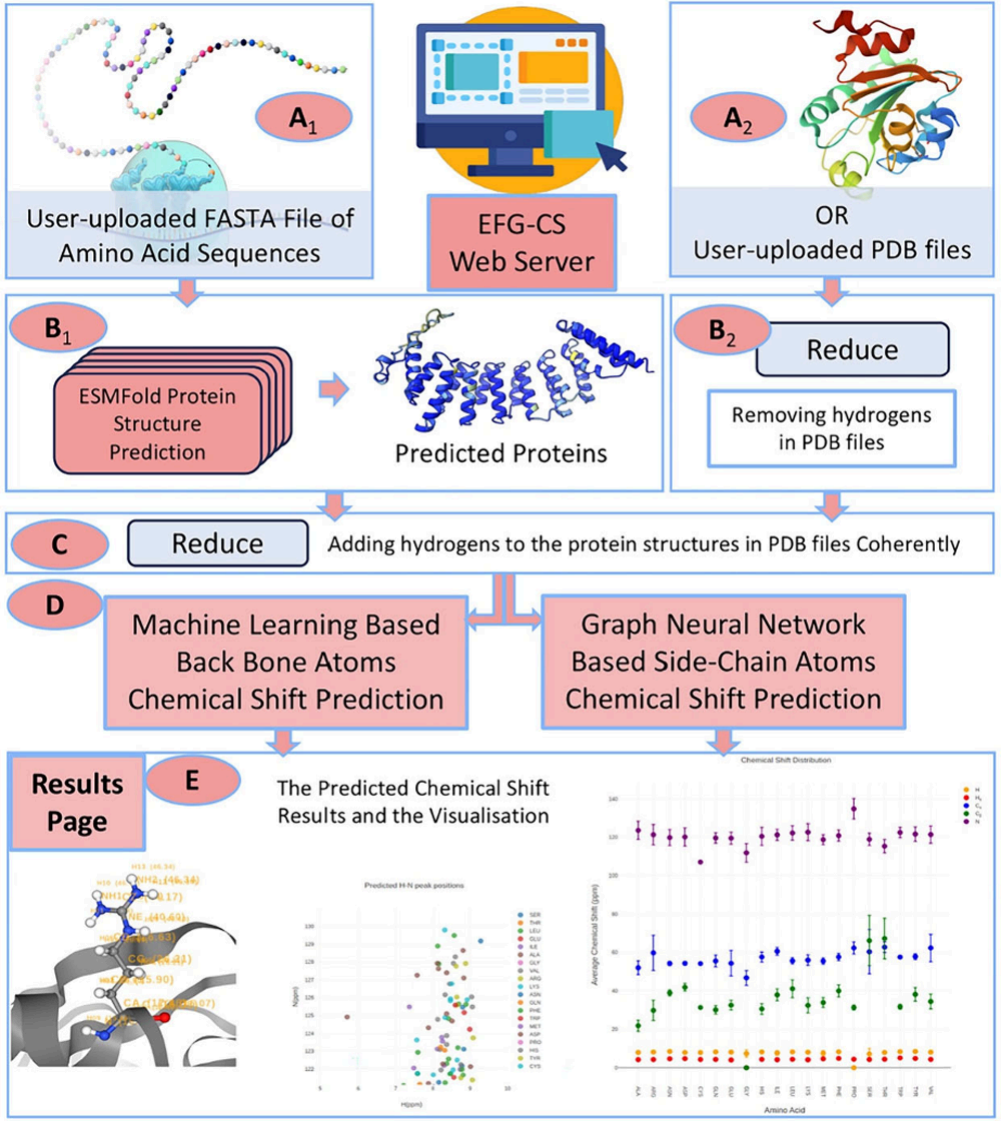
Computational pipeline of the EFG-CS model for predicting protein chemical shifts. (a) The user inputs amino acid sequences or protein structures. (b) Protein structure prediction using ESMFold (for sequences) or preprocessing uploaded structures. (c) Hydrogen atom addition to predicted or uploaded structures. (d) Machine learning and graph neural network-based predictions of backbone and side-chain chemical shifts, respectively. (e) Visualization and downloadable chemical shift outputs via the EFG-CS Web server. Reproduced with permission from reference 409. Copyright (2024) by Wiley Periodicals LLC on behalf of The Protein Society under the Creative Commons Attribution (CC BY) license.

Additional performance evaluations were carried out using the SHIFTX2 test data set^411^ for side-chain atom predictions, with EFG-CS achieving RMSEs ranging from 0.58 ppm (*Hγ*_2_) to 1.26 ppm (*Hε*_2_). These values, obtained without data curation or manual preprocessing, reflect the model’s strong generalizability and alignment with realistic application environments. However, the results also highlight remaining challenges particularly in modeling structurally flexible residues, disordered regions, or post-translational modifications, where broader RMSE distributions were observed. To further enhance prediction accuracy, future development may focus on improving long-range electrostatic modeling, refining treatment of solvent accessibility, and incorporating uncertainty quantification within deep learning architectures. The convergence of end-to-end protein structure prediction with chemical shift estimation in EFG-CS exemplifies the accelerating shift toward integrated learning-based models that advance both mechanistic insight and translational discovery in NMR.

The evolution of machine learning in structural biology has been marked by a shift from broad-spectrum predictors to models tailored to the hierarchical and dynamic complexity of proteins. Among these developments, a model called LEGOLAS (neuraL nEtwork enGine fOr caLculating chemicAl Shifts) was introduced by Darrows et al.^114^ This model was designed using the TorchANI framework. It has been optimized for the rapid and accurate prediction of protein NMR chemical shifts. A critical challenge in the field, the need for fast, differentiable, and accurate models across large structural ensembles has been addressed by LEGOLAS, particularly for systems encountered during MD simulations. By encoding atomic environments through atomic environment vectors (AEVs) derived from symmetry functions, LEGOLAS circumvents the limitations of discrete structure encodings. The model supports end-to-end GPU acceleration and differentiability, thereby enabling direct integration into refinement protocols and large-scale dynamic sampling pipelines. When benchmarked against SHIFTX2,^411^ LEGOLAS achieved similar or better accuracy across key nuclei (e.g., *C*_*α*_,*C*_*β*_, *H*_*α*_) with a 10-fold increase in computational speed. Its ensemble architecture of five independently trained neural networks contributed to improved generalization and noise robustness. Importantly, this approach enables time-averaged prediction across MD snapshots-a capability that remains largely unmet in earlier methods. The model architecture and deployment pipeline are schematically depicted in Figure 12. Thus, LEGOLAS represents a critical advancement by directly targeting both the accuracy and scalability requirements needed for real time, structure-aware NMR modeling in proteins.

**Figure 12:**
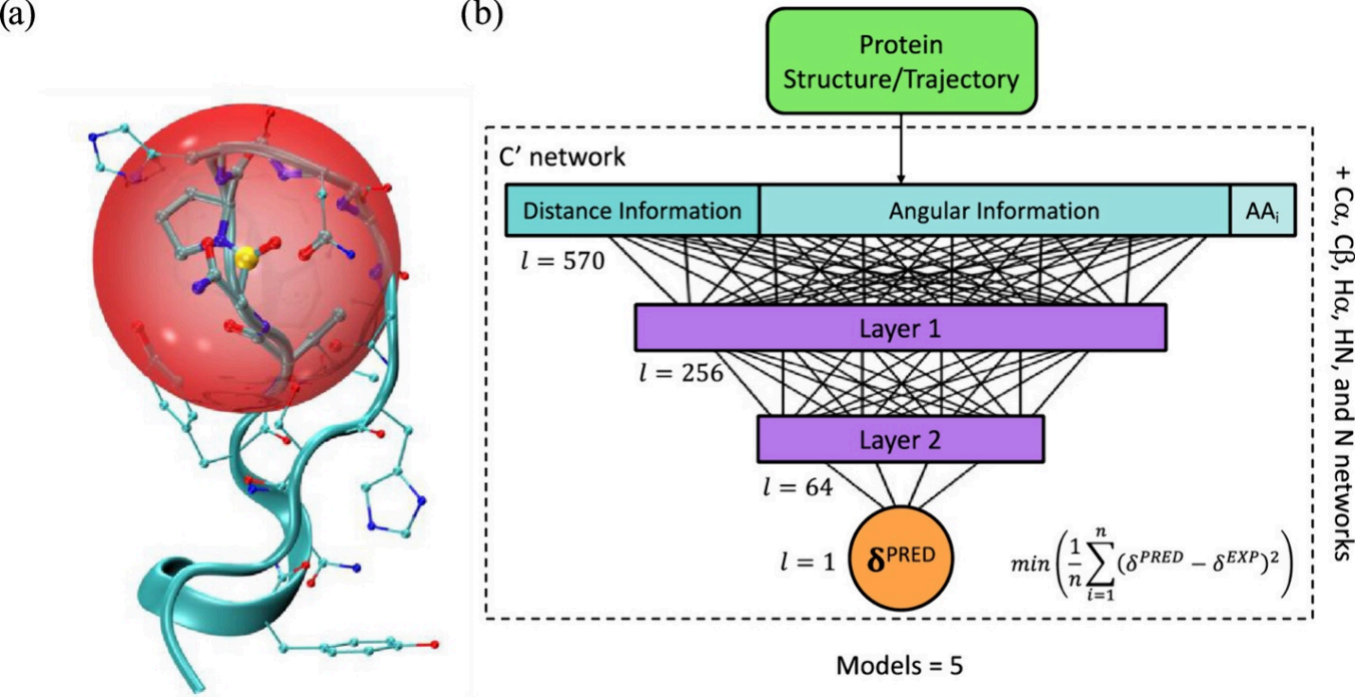
(a) Representation of the atomic environment vector (AEV) radial cutoff sphere (5.1 Å) centered on a carbonyl carbon, illustrating the spatial encoding of neighboring atoms within the LEGOLAS framework. (b) Schematic of the LEGOLAS neural network architecture for protein NMR chemical shift prediction. Input protein structures or trajectories are processed into atom-specific AEVs incorporating radial and angular distance metrics, as well as residue-type embeddings. Each AEV, of length 570, is passed through two fully connected hidden layers (256 and 64 neurons, respectively) with exponential linear unit (ELU) activation functions, culminating in the predicted chemical shift output (δ^PRED^). An ensemble of five independently trained networks per backbone atom type is employed for robust prediction. The model is optimized by minimizing the mean squared error between predicted and experimental shifts. Reproduced with permission from reference 114. Copyright (2025) American Chemical Society.

Compared to other contemporary ML models for NMR chemical shift prediction, LEGOLAS distinguishes itself through its atom-wise architecture and systematic handling of protein structure as radial and angular atomic environment vectors (AEVs). In contrast to CASCADE, which applies graph neural networks to small organic molecules by learning spectral shifts through message passing among molecular graph nodes, LEGOLAS encodes detailed spatial and chemical features via predefined AEVs and performs independent predictions for each backbone atom type, enhancing modularity and interpretability. The use of multiple independently trained networks, one for each atom class, has been shown to improve robustness across varying residue types. While PROSPRE^344^ integrates equivariant neural networks with ESM-derived embeddings for sequence-informed protein structure encoding, its reliance on large protein language models introduces additional computational cost and complexity. By comparison, LEGOLAS leverages direct structural inputs from PDB or trajectory files without sequence-based pretraining, achieving competitive accuracy for ^1^H, ^13^C, and ^15^N shifts. Furthermore, unlike the NMR-GNN^403^ framework, which incorporates attention mechanisms over chemical bond types and 3D geometry for general-purpose shift prediction across atom types, LEGOLAS maintains a biologically specialized design tailored to protein backbone prediction. This focused scope facilitates more efficient training and accurate reproduction of experimental chemical shifts, particularly in structured protein regions, and demonstrates the value of atom-wise modular design in biomolecular NMR modeling.

To address the fragmentation of prediction strategies across liquid-state and solid-state NMR applications, Xu et al.^412,413^ proposed a unified ML framework called NMRNet, which leverages a two-stage approach involving large-scale pretraining and task-specific fine-tuning. The motivation behind NMRNet stems from a pressing need for models that can operate across diverse chemical domains, ranging from small organic molecules in solution to periodic solids in materials chemistry, without sacrificing either speed or accuracy. The framework, schematically illustrated in Figure 13, consists of four main modules including data preparation, large-scale structural pretraining, supervised fine-tuning on NMR data sets, and flexible inference modes for spectral analysis. During pretraining, over 4.8 million 3D structures were utilized to learn robust atomic environment representations, mitigating limitations imposed by sparse experimental NMR data. For the fine-tuning stage, two curated data sets for solution phase spectra and ShiftML^414^ for solid state predictions were used to optimize the model for different applications. The result is a system capable of achieving state-of-the-art accuracy across multiple elements and chemical systems. In liquid-state testing, NMRNet reduced the mean absolute error for ^1^H and ^13^C shifts to 0.181 and 1.098 ppm, respectively, even when applied to molecules with over 150 atoms. In solid-state cases, the model demonstrated strong correlation with DFT-derived values for elements such as ^15^N and ^17^O. These benchmarks highlight the model’s generalization capacity and set a new standard for cross domain chemical shift prediction. Importantly, NMRNet was designed not only for numerical prediction but also for advanced inference tasks such as peak assignment and configuration determination, including stereoisomer resolution in chiral systems. The ability to handle such tasks without requiring extensive domain specific feature engineering or hand-curated rule sets marks a significant methodological advancement in the use of deep learning for NMR interpretation.

**Figure 13:**
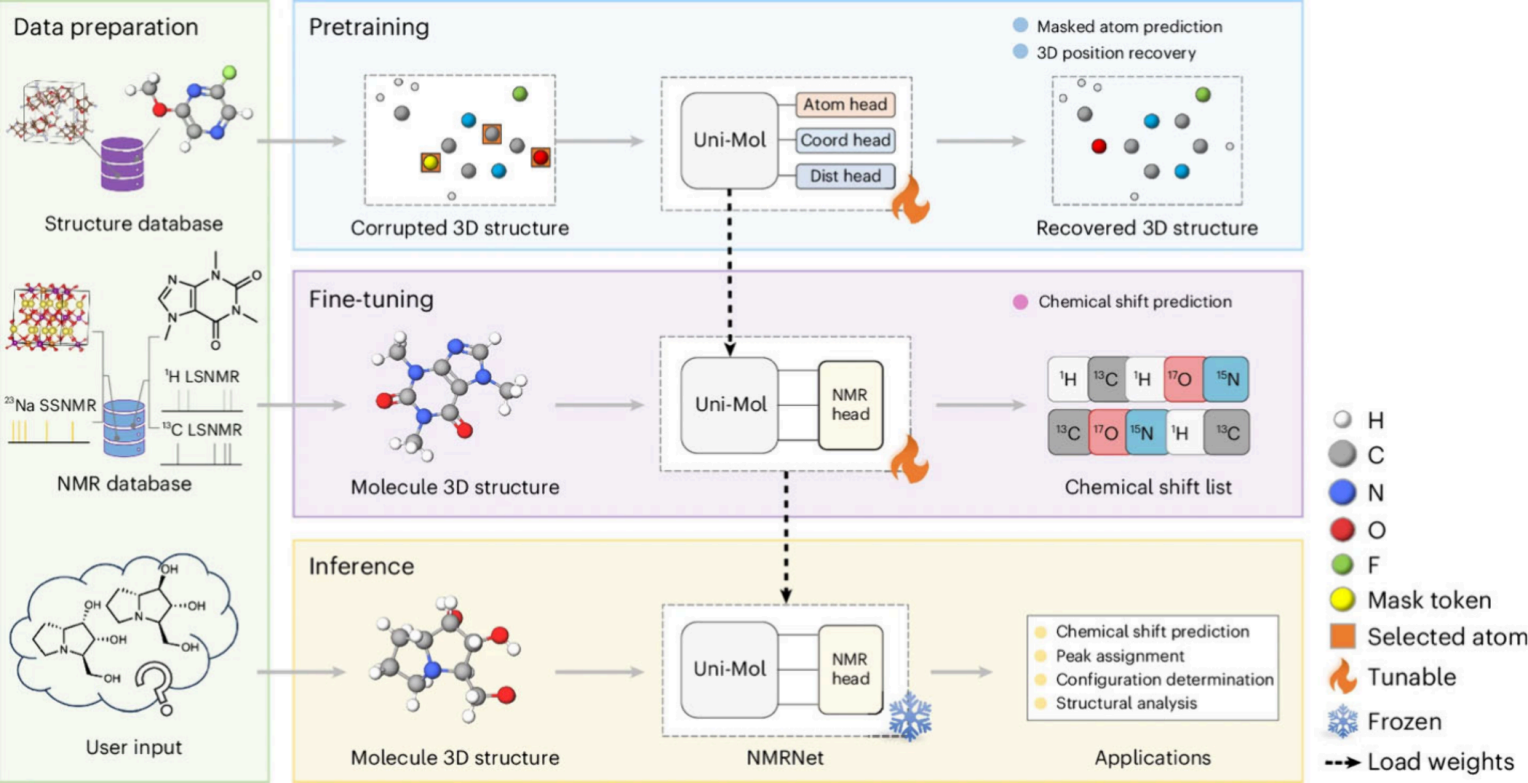
Schematic representation of the NMRNet framework for chemical shift prediction. Left: data preparation pipeline integrating structural information from both liquid-state (LSNMR) and solid-state NMR (SSNMR) sources. Right top: self-supervised pretraining using molecular structures to perform masked atom type recovery and 3D coordinate reconstruction. Right middle: supervised fine-tuning phase leveraging annotated NMR chemical shift data sets. Right bottom: inference phase in which pretrained and fine-tuned model weights are applied to downstream prediction tasks. Coord = coordinate and Dist = distance. Adapted from Xu et al., arXiv:2408.15681v1,^413^ Copyright (2025), under a Creative Commons CC BY 4.0 license.

In a critical assessment of its performance and limitations, Xu et al. conducted several validation experiments to explore the boundaries of NMRNet’s predictive capacity and its alignment with structural complexities. In fine-tuned applications involving nerve agents and high-molecular-weight compounds, the model maintained R^2^ values above 0.95 for both ^1^H and ^13^C shifts, confirming that predictive accuracy was retained across increasing molecular size. When benchmarked against previous models on the QM9-NMR^415^ and nmrshiftdb2 data sets,^416^ NMRNet achieved a reduction in mean absolute error from 0.054 to 0.020 ppm for ^1^H and from 0.520 to 0.262 ppm for ^13^C, demonstrating clear improvements over established baselines. In the domain of peak assignment, a 94% accuracy rate was obtained for ^13^C NMR in nerve agent molecules, although performance for ^1^H lagged at 72%, revealing challenges related to signal crowding and resonance overlap. One of the key unresolved challenges is the model’s limited ability to generalize solvation effects and temperature induced chemical shift variability,^417^ which are often critical in experimental settings but poorly captured in available training data. Additionally, stereochemical discrimination in chiral systems remains partly dependent on dual nucleus inputs, highlighting a need for improved single-nucleus resolution strategies. Xu et al. also emphasized that performance gains were largely attributable to large-scale pretraining, particularly in data-sparse regimes, suggesting future directions involving curated multimodal data sets, integration with quantum mechanical descriptors, and incorporation of domain adaptation techniques. Therefore, NMRNet serves as both a functional tool for current NMR modeling and a template for next-generation ML pipelines aimed at comprehensive spectrum-structure correlation across chemical systems. Its introduction represents a critical step toward solving the long-standing problem of generalizability in NMR prediction and contributes essential insights into the design of models that are simultaneously accurate, scalable, and chemically informed.

An especially impactful advancement in ML-augmented NMR modeling is the DU8ML framework developed by Novitskiy and Kutateladze.^418^ DU8ML is a hybrid protocol that builds upon the DU8+ method by combining lower-level DFT calculations with machine-learned substructure-specific correction terms to significantly enhance accuracy and throughput in predicting ^13^C chemical shifts and proton–proton spin–spin coupling constants. Trained on over 11000 experimental ^13^C shifts and 4000 J-couplings primarily in chloroform, DU8ML achieves RMSDs of approximately 0.95 ppm for ^13^C and 0.28 Hz for ^1^H–^1^H J values, enabling rapid and reliable validation of large natural products and alkaloids. The approach successfully revised 35 misassigned structures from a test set of 170 alkaloids, demonstrating its practical utility in resolving common challenges such as heavy atom misassignments, epimerization errors, and N-oxide stereochemistry. The DU8ML protocol and model architecture are schematically illustrated in Figure 14. Notably, DU8ML bypasses the need for exhaustive spin–orbit coupling calculations by applying substructure-aware empirical corrections, providing speed and accuracy without resorting to expensive relativistic methods. With typical computation times under 20 min per conformer on standard HPC nodes, DU8ML offers a robust solution for high-throughput structure verification and reassignment in complex natural product discovery pipelines. One of the key remaining challenges is extending the model’s applicability beyond alkaloid and natural product scaffolds, particularly for flexible systems, varied solvents, and heteroatom-rich environments not well represented in the training set.

**Figure 14:**
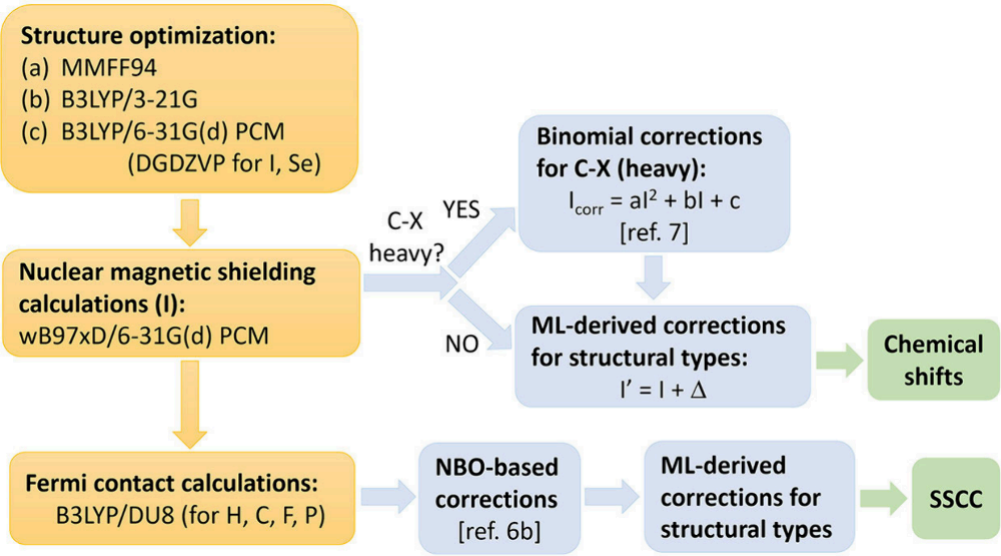
Workflow of the DU8ML protocol for high-throughput NMR chemical shift and scalar spin–spin coupling constant (SSCC) prediction. Orange modules denote quantum chemical calculations performed at various levels of theory, including structure optimization shielding constant evaluation and Fermi contact calculations. Blue modules represent empirical corrections, including binomial corrections for heavy atom-bearing carbons (C-X), NBO-based corrections, and ML-derived corrections based on substructure types. These refinements yield chemically accurate predictions of NMR observables with minimal computational cost. Reproduced with permission from reference 418. Copyright (2022) American Chemical Society.

While models like DU8ML have demonstrated remarkable accuracy in small-molecule and natural product prediction through hybrid QM-ML strategies, recent efforts in protein NMR have shifted toward integrating high-resolution structural features with learning-based frameworks. One such advancement is UCBShift 2.0,^419^ which introduces a modular, physics-aware approach to predicting protein chemical shifts across diverse backbone and side-chain environments. Developed by Ptaszek et al., UCBShift 2.0 fuses two complementary components: UCBShift-X, which uses a comprehensive set of physics-informed structural features derived from protonated X-ray structures, and UCBShift-Y, which applies transfer learning from homologous proteins using TM-align-based structural alignment. These modules are integrated into a multistage random forest regression model that predicts both backbone and side chain chemical shifts, offering flexibility in cases where homologous templates are available. As depicted in Figure 15, the model was trained on 851 protein structures from the RefDB database paired with BMRB chemical shift data, and evaluated on an independent test set of 200 proteins. UCBShift 2.0 achieved mean absolute errors of 0.80 ppm for ^13^C, 0.16 ppm for ^1^H, and 0.99 ppm for ^15^N atoms in side chains, outperforming SHIFTX2 ^411^ across all atom types. In particular, the model demonstrated improved accuracy for atom environments that are traditionally difficult to predict, such as isoleucine CD1, where conformational variability due to $X_{2}$ rotamers and, in other cases, π-stacking interactions significantly influence chemical shifts.

**Figure 15:**
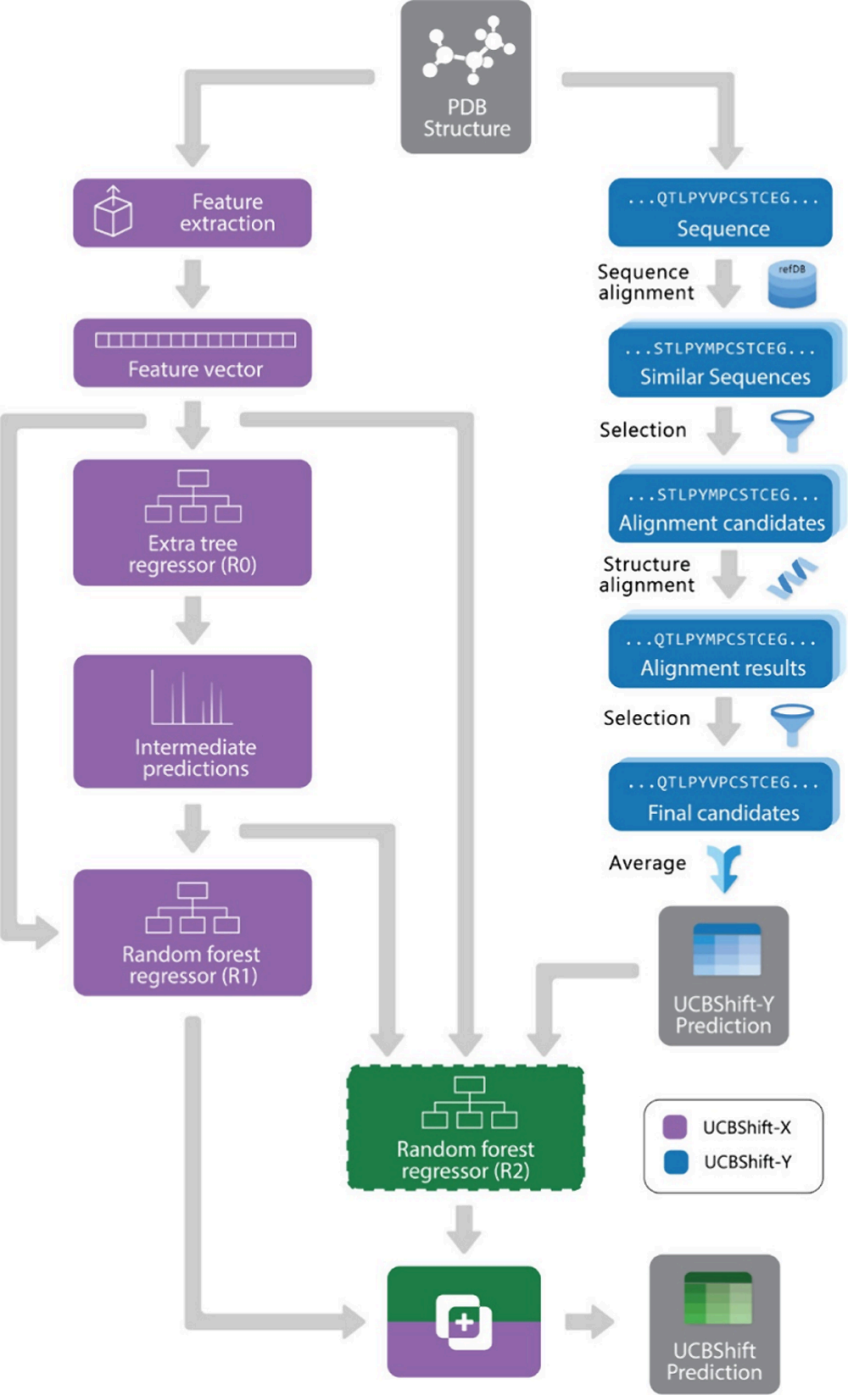
Schematic workflow of the UCBShift model for protein chemical shift prediction. The UCBShift framework integrates two complementary modules: UCBShift-X (left path), a feature-based ML pipeline employing extra tree and random forest regressors trained on structural descriptors extracted from X-ray protein structures and UCBShift-Y (right path), a transfer-learning module that identifies homologous sequences and structures from a reference database to assign chemical shifts via similarity weighted averaging. The final predictions are produced by a combined random forest model that integrates outputs from both modules, enhancing accuracy across both backbone and side chain atom types. Reproduced with permission from reference 419. Copyright (2024) American Chemical Society.

Critically, the improved performance of UCBShift 2.0 stems from both its refined input representations and its hybrid learning strategy. Features such as backbone and side chain dihedral angles, hydrogen bond counts, B-factors, solvent accessibility, and local electric field vectors contribute to the model’s chemical sensitivity. The TM-align based homology module enables conditional predictions tailored to structural analogs, contributing to a 0.5 and 0.2 ppm improvement in ^13^C and ^1^H shift accuracy, respectively, over the standalone X-module. The model was also able to resolve protonation-state dependent chemical shift perturbations in charged residues, such as histidine and glutamine, which are difficult to capture using simpler backbone-focused models. The open-source implementation and modular design of UCBShift make it particularly suited for large-scale protein NMR analysis, ensemble refinement, and real-time assignment validation. One of the key unresolved challenges is the model’s limited nucleus coverage, especially for nonstandard side chains and metal-binding environments, which restricts its applicability in metalloproteins and complex cofactors. Future efforts will benefit from expanding training sets to include a broader diversity of structural motifs, improving nucleus-specific parametrization, and incorporating dynamic ensemble averaging to further enhance prediction accuracy in flexible or partially disordered regions.

The interpretive bottleneck in structure elucidation often lies not in spectral acquisition but in resolving subtle spectroscopic distinctions among stereoisomers and constitutional analogues. To address this longstanding challenge, Marcarino et al.^103,420^ developed the Artificial Neural Network-Pattern Recognition Algorithm (ANN-PRA), a data-driven method designed to systematically correlate theoretical and experimental NMR chemical shifts for robust structure verification. The protocol combines artificial neural networks with unsupervised clustering and chemometric scoring to rank candidate structures based on shift agreement, while accounting for solvent effects and conformational variability. By mapping input features such as weighted statistical shift errors and chemical environment fingerprints, ANN-PRA learns patterns that distinguish between correct and incorrect molecular connectivities, even when traditional error metrics like mean absolute deviation or DP4 probability produce ambiguous results.^421^ In benchmark tests involving a range of organic compounds, including stereoisomers with near-identical ^1^H and ^13^C shift patterns, ANN-PRA demonstrated a marked improvement in false-positive reduction, correctly identifying erroneous proposals in cases where DFT-based shift matching alone failed. Furthermore, the method is solvent-aware, enabling improved ranking accuracy across polar and nonpolar media by dynamically adjusting shift interpretation using reference data sets. The overall structure of the ANN-PRA workflow is illustrated in Figure 16. The model’s explainability was enhanced by saliency maps, which highlight the most chemically influential atoms in driving structure discrimination, offering insight into NMR-descriptor relevance within the network.

**Figure 16:**
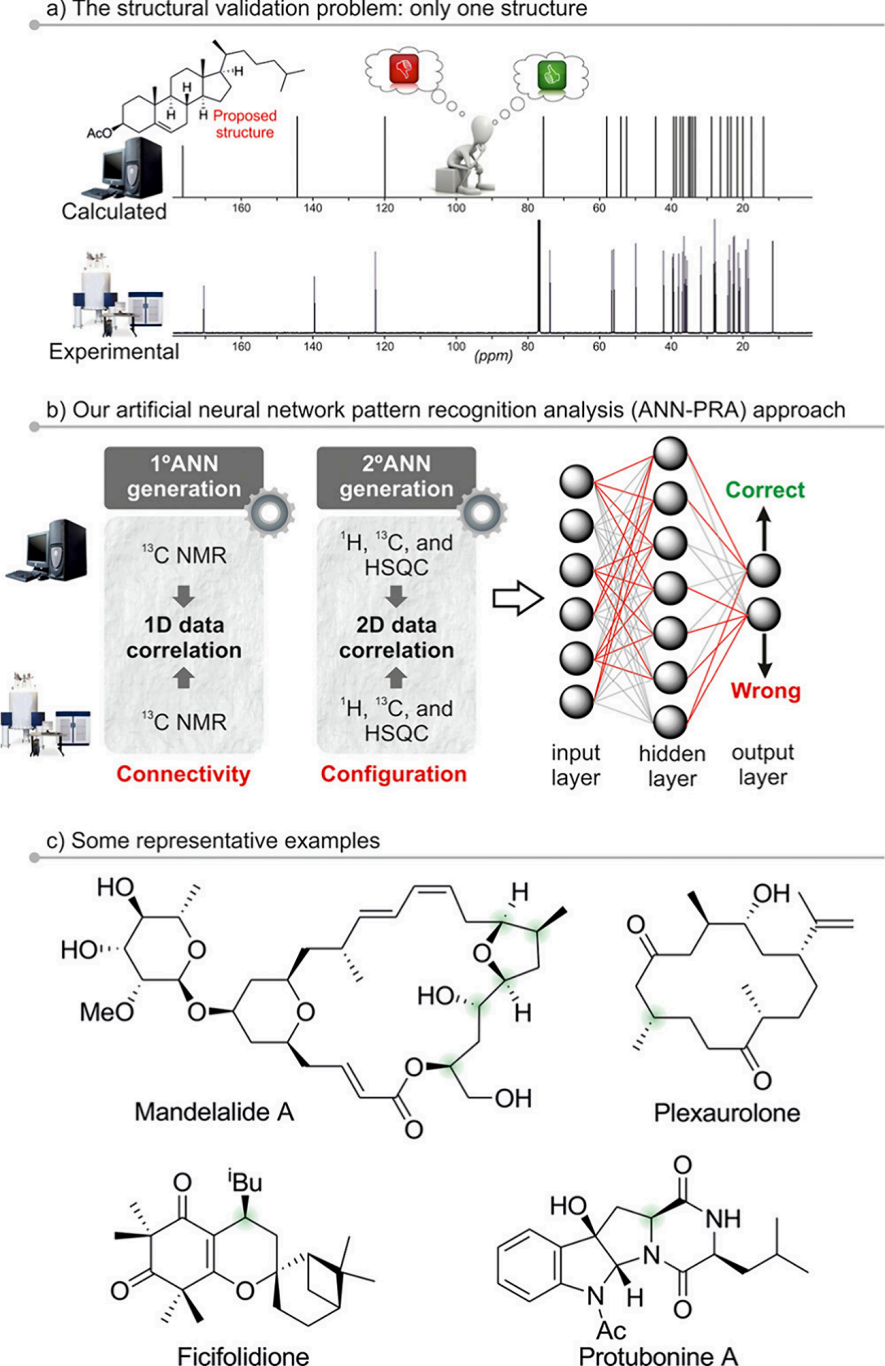
Overview of the ANN-PRA framework for structural validation using NMR. (a) Illustration of the structure validation problem where only a single proposed structure is available and must be evaluated against experimental data. (b) Schematic of the ANN-PRA architecture, which applies a two-stage neural network system: the first trained on 1D ^13^C NMR correlations to assess atom connectivity, and the second using combined ^1^H, ^13^C, and HSQC 2D data to evaluate stereochemical configurations. (c) Examples of natural products that were originally misassigned, with stereocenters requiring revision indicated by green dots. Reproduced with permission from reference 103. Copyright (2020) American Chemical Society.

Beyond its standalone utility, ANN-PRA offers a blueprint for hybridizing pattern recognition with QM data in high-throughput structure validation pipelines. The approach is particularly effective for detecting misassigned connectivities and regioisomeric confusion in systems where stereochemistry plays a minimal role but structural ambiguity remains high. One of the key challenges identified by Sarotti and co-workers is the diminished predictive confidence when trained models are applied to highly strained or nonbenzenoid scaffolds outside the chemical domain of the training set. The authors propose that model robustness may be further improved by integrating fragmentation-based alignment procedures and by retraining networks using curated error prone data sets with known experimental misassignments. Another promising direction involves coupling ANN-PRA outputs with automated spectral assignment tools to enable end-to-end decision-making in spectroscopic workflows.^422,423^ As machine learning continues to redefine how chemists interpret NMR data, methods like ANN-PRA underscore the growing importance of feature engineering, domain transferability, and model transparency in the deployment of predictive NMR analytics. These innovations pave the way for more intelligent, interpretable, and context-aware structure verification systems capable of operating across broad chemical and spectral domains.

In pursuit of fully autonomous stereochemical elucidation, Howarth et al.^422^ introduced DP4-AI, a high-throughput, open-source platform that automates the entire NMR structure validation workflow, encompassing spectral processing, chemical shift prediction, assignment, and configurational probability scoring. The motivation stems from limitations in traditional DP4 methods, which rely on manual spectrum interpretation and are thus prone to user bias, limited scalability, and low throughput. DP4-AI replaces these steps with automated modules for Fourier transformation, phasing, solvent peak identification, baseline correction, and multiplet deconvolution, followed by probabilistic structure ranking using the Bayesian DP4 framework. The full architecture is depicted in Figure 17. When evaluated on a test set of 47 chemically diverse molecules including 21 natural products, 10 synthetic intermediates, and 16 stereochemically complex scaffolds the system correctly identified the correct isomer in 43 out of 47 cases, yielding an overall assignment accuracy of 91%. Importantly, 16 of these molecules contained four or more stereocenters, and 10 included flexible rings or acyclic segments where conformational averaging complicates shift prediction. For the natural product NP2, a particularly challenging 10-membered macrolide with six stereocenters, DP4-AI assigned the correct structure from a pool of 64 possible diastereomers using only ^1^H and ^13^C 1D spectra in under 12 min. These performance metrics mark a significant leap in both accuracy and automation compared to earlier semiautomated methods that required days of human input. Moreover, spectral referencing and assignment steps are dynamically optimized by internal confidence metrics, allowing DP4-AI to adjust to noisy or partially resolved experimental data. Despite these successes, one of the key unresolved challenges is the treatment of conformationally dynamic systems, particularly those with multiple low-energy conformers that contribute nonlinearly to averaged NMR shifts. Current GIAO-based predictions do not sufficiently capture this complexity without extensive conformational sampling, which remains computationally intensive and often inaccurate in solvent-sensitive systems. The current implementation assumes monocomponent mixtures and performs less reliably on overlapping multiplets or mixture spectra. The authors highlight promising future directions including the incorporation of 2D NMR data sets, improved conformational sampling algorithms, and a generalization of the Bayesian model to account for correlated prediction errors across nuclei. DP4-AI not only delivers an end-to-end automated solution for routine NMR-based stereochemical assignment but also establishes a critical foundation for future developments that couple experimental spectra with statistically grounded quantum predictions at scale.

**Figure 17:**
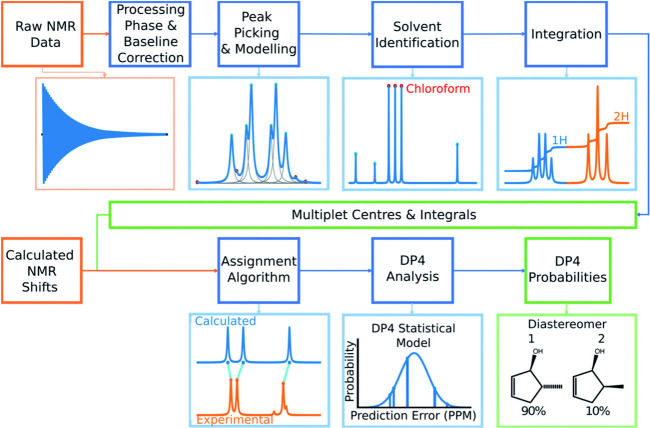
Schematic workflow of the DP4-AI framework for automated structure validation using raw 1D NMR spectra. The pipeline begins with raw spectral data, which undergoes automated phase correction, baseline adjustment, peak picking, solvent identification, and integration to extract multiplet centers and integrals. These features are matched to DFT-calculated NMR shifts using an assignment algorithm, followed by DP4 statistical analysis to yield stereochemical probabilities. The system enables fully automated, high-throughput diastereomeric discrimination with minimal user intervention. Reproduced with permission from reference 422. Copyright (2020) The Royal Society of Chemistry.

Building upon the automation framework of DP4 AI, Howarth and Goodman^424^ introduced the DP5 probability model to tackle a critical gap in structure verification, namely the absence of statistical rigor when only a single structure candidate is available. While DP4 assumes the correct structure is present in a set of diastereomers, DP5 abandons this constraint by quantifying the absolute probability that a proposed structure is correct, based solely on ^13^C NMR data and DFT calculated shifts. The DP5 workflow is depicted in Figure 18. In this framework, atomic-level prediction errors extracted from over 63000 DFT-computed NMR shift deviations across a reference set of 5140 molecules are processed using Gaussian kernel density estimation to construct atom-specific probability distributions. These atomic probabilities are then combined using a Boltzmann weighted geometric mean and calibrated through Bayesian correction to yield a normalized molecular level probability. When benchmarked on 13 real world structure reassignments from the literature, DP5 assigned near zero probabilities to all initially incorrect structures and significantly higher confidence to their correct revisions, demonstrating its practical value in detecting subtle errors in stereochemistry or substitution. In a cross validation test designed to match correct and incorrect spectra with equivalent DFT mean absolute errors of approximately 1.6 ppm, DP5 maintained its discriminatory power, underscoring its ability to distinguish candidates that would otherwise confound an expert chemist. One major challenge is posed by the intrinsic accuracy limits of DFT, which restrict the maximum DP5 probability to 72% even for correct structures. This ceiling is caused by uncertainties in atomic environments and DFT functional errors. Future DP5 versions are expected to include multidimensional NMR data, better conformer handling, and integration with generative design workflows. By offering a mathematically grounded, structure-independent confidence score, DP5 is positioned to enhance NMR analysis for cases where a single structure must be evaluated in isolation.

**Figure 18:**
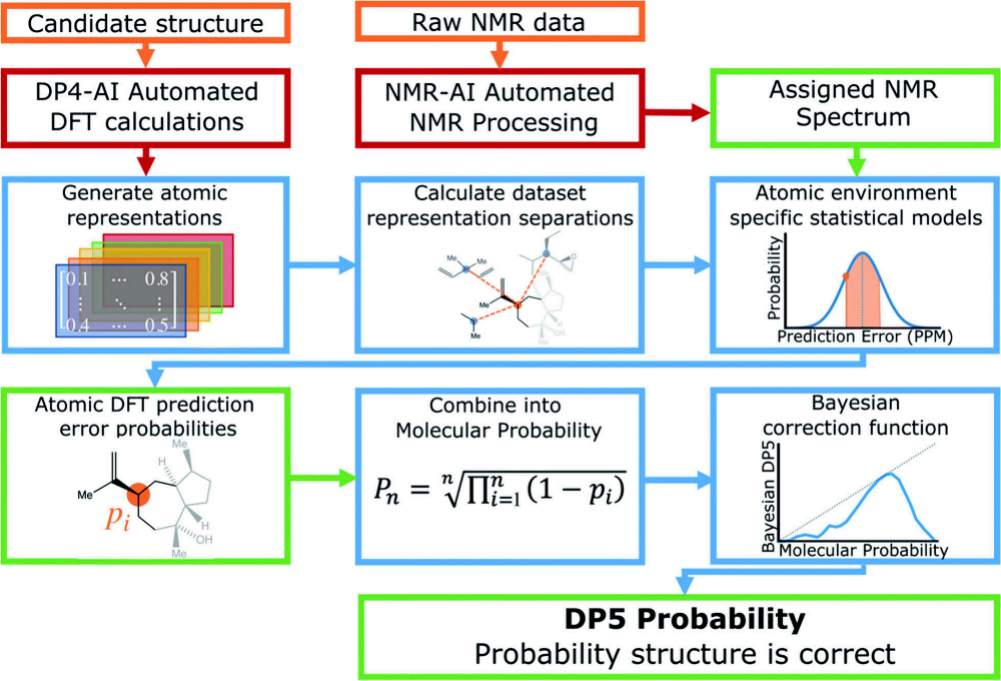
Schematic overview of the DP5 probability framework for single-structure validation using ^13^C NMR data. Starting from a candidate molecular structure and raw NMR spectrum, the pipeline integrates automated DFT shift prediction (via DP4-AI) with automated spectral processing (via NMR-AI). Atomic representations are used to quantify DFT prediction error probabilities using atom-environment-specific statistical models. These probabilities are aggregated into a molecular-level probability and refined through a Bayesian correction function to yield the final DP5 probability, which represents the likelihood that the proposed structure is correct. Reproduced with permission from reference 424. Copyright (2022) The Royal Society of Chemistry.

In addition to the models discussed in detail above, several other ML methods have been developed that extend the applicability of NMR predictions across chemical and biomolecular domains. A diverse range of ML models has emerged in recent years for predicting NMR parameters, each tailored to specific molecular classes, nuclei, or spectral types. Notable among these is IMPRESSION (IMPRESSION-G2),^113,425^ which leverages kernel ridge regression trained on DFT-level data to predict ^1^H, ^13^C chemical shifts, and ^1^J_CH_ scalar couplings with near-quantum accuracy for organic molecules. Similarly, the MPNN-NMRML approach^426^ has deployed ensembles of message-passing neural networks to enhance ^13^C chemical shift predictions in small molecules, with a strong emphasis on data set diversity and uncertainty quantification. ShiftML^414^ has focused on crystalline materials, particularly for solid-state NMR, using Gaussian approximation potentials and symmetry-aware atomic descriptors to predict shielding tensors. Expanding to protein-scale predictions, models such as PLM-CS (protein-language-model-based chemical shift prediction)^427^ integrate protein language models with downstream neural networks to predict backbone shifts without requiring explicit structure inputs, while EFG-CS^409^ combines ESMFold-generated^428^ structures with hybrid models transfer learning for backbone atoms and GNNs for side chains to enable full-protein chemical shift prediction pipelines.

Beyond these, additional models have been introduced to address niche applications and specialized NMR modalities. The GT-NMR framework,^429^ built on graph transformers, has reported state-of-the-art performance on small-molecule ^1^H and ^13^C shift prediction, whereas TransPeakNet^430^ targets HSQC data sets from HMDB 5.0 ^431^ using multitask deep learning to resolve cross-peaks and correlation patterns. Solid-state-focused models include Solid-state GNN,^432^ which predicts shielding tensors such as those of SiO_2_, and ^17^O-specific predictors that utilize molecular descriptors for oxide environments. Specialized efforts for zeolites have yielded ML models^433^ for ^27^Al shift prediction, conditioned on framework type and extrinsic factors. Importantly, tools like 2DNMRGym^434^ have also emerged as curated data sets that accelerate the training of ML models on 2D NMR spectra. Meanwhile, earlier statistical or empirical approaches, SHIFTX2,^411^ SPARTA+,^435^ CamShift,^436^ and PROSHIFT^437^ pioneered the domain of protein chemical shift prediction by combining torsion angle potentials, empirical corrections, and chemical environment descriptors, though they have now largely been supplanted by neural and hybrid architectures that better accommodate structural flexibility and residue interactions. More recently, the MIM-ML^379^ model has been developed as a fragment-based random forest approach trained exclusively on DFT-derived data for nucleic acids, enabling accurate prediction of both ^1^H and ^13^C chemical shifts without experimental fitting. These developments illustrate the accelerating momentum and increasing granularity with which ML models are addressing the multifaceted challenge of NMR shift prediction.

## 4 NMR Shifts in QSAR Modeling

NMR chemical shifts have long served as molecular descriptors in QSAR studies, providing insights into electronic and steric influences on biological activity. Foundational work by Pople and Karplus established correlations between chemical shifts and molecular geometry, enabling their application in predicting physicochemical properties and biological activity. Integration of ^13^C and ^1^H NMR shifts into QSAR models has enhanced drug design, toxicity assessments, and bioactivity predictions, supported by advancements in quantum chemical calculations for complex systems.^438–441^ The incorporation of machine learning, including graph neural networks, has further refined shift predictions and improved QSAR model performance. Computational frameworks such as CS-ROSETTA and CS23D have bridged NMR data with predictive modeling, streamlining structural elucidation and cheminformatics applications.^442,443^

The application of ^13^C chemical shifts in QSAR has demonstrated correlations with electronic parameters such as Hammett substituent constants (σ, σ^+^ ,σ), atomic charges, and frontier molecular orbitals (HOMO, LUMO). Chemical shifts of substituent-bearing carbons often correlate directly with substituent electronic properties, making them useful predictors of electronic interactions in molecules. Downfield chemical shifts are typically associated with electron-withdrawing substituents, while electron-donating substituents produce upfield shifts. This relationship has facilitated numerous studies involving structure–activity predictions for diverse biological end points, such as antimicrobial, antiviral, and anticancer activities, as well as reactivity and stability assessments.^128,444–446^

Quantitative Spectrometric Data–Activity Relationship (QSDAR) methodologies^447^ explicitly integrate chemical shift data into QSAR models, enhancing their predictive capabilities. Techniques like Comparative Structural Connectivity Spectra Analysis (CoSCoSA)^448^ combine structural and spectral data, providing deeper insight into the relationship between chemical structure and biological activity. The combination of chemical shifts with classical molecular descriptors-including electronegativity, polarizability, bond dissociation energies, and ionization potentials-further strengthens the predictive power of QSAR models. These integrated approaches have been effectively employed to predict enzyme inhibition, receptor–ligand interactions, environmental persistence, and compound toxicity.^449–451^

The evolution of QSAR modeling with NMR data continues to be driven by high-throughput spectral databases and AI-driven methodologies, underscoring the critical role of chemical shifts in modern cheminformatics.^452^ Rapid advancements in computational chemistry and machine learning have significantly accelerated the ability to interpret complex spectral data, allowing extensive QSAR model training and validation against large chemical data sets. These developments highlight the ongoing importance of NMR chemical shifts as fundamental descriptors in QSAR, paving the way for enhanced molecular design and predictive accuracy in pharmaceutical, environmental, and chemical research.

## 5 Challenges and Future Prospects

Computational NMR methodologies, encompassing quantum chemical calculations, machine learning predictions, and QSAR techniques have advanced significantly over the years yet persistent challenges remain. Quantum chemical NMR calculations, often utilizing density functional theory, encounter inherent limitations related to electron correlation, relativistic effects, and the static approximation of molecular structures. Although quantum mechanical NMR computations can predict molecular structures with high accuracy, they are computationally expensive and become prohibitively costly as system size increases. Additionally, DFT frequently fails to capture the temporal evolution of solvation structures under varying conditions such as temperature, pressure, pH, and composition. These computational approaches often struggle to accurately reflect solvation effects, rovibrational dynamics, and conformational flexibility, factors essential for precise chemical shift predictions. Consequently, developments in advanced sampling methods, polarizable force fields, and explicit relativistic corrections remain critical to overcome these intrinsic computational limitations.

Machine learning has emerged prominently within computational NMR, offering significant reductions in computational time compared to classical quantum methods. However, ML-based methods require extensive, high-quality data sets for effective training, presenting a substantial bottleneck due to the limited availability and chemical diversity of current data sets. Techniques such as ShiftML,^453^ IMPRESSION,^113^ CASCADE,^112^ and ML-J-DP4 ^115^ demonstrate that ML models trained on DFT-generated chemical shifts can achieve high predictive accuracy with drastically reduced computational costs, although these methods remain dependent on the quality of input structures. Future directions necessitate expanding training data sets, refining algorithms for accuracy, and enhancing interpretability to clarify underlying physicochemical relationships.

In the QSAR domain,^128^ significant challenges arise from the complexity of spectral data analysis and the difficulty in reliably extracting meaningful quantitative descriptors from NMR spectra due to spectral overlap and variations caused by experimental conditions. QSAR models must also robustly integrate spectral data with physicochemical and omics data. Improved statistical methods and data preprocessing techniques capable of accurately parsing complex spectral data sets will substantially enhance QSAR’s applicability and reliability.

Looking ahead, one of the most promising frontiers in computational NMR lies in the development of robust machine learning models trained on quantum-derived data sets. Unlike many analytical modalities that are limited by sparse or inconsistent experimental data, NMR provides a pathway to systematically generate large, chemically diverse, and physically grounded data sets using first-principles methods. This makes it possible to construct predictive models without requiring massive experimental campaigns. However, challenges remain in reconciling computed and experimental data, especially in capturing effects such as solvation, conformational dynamics, and instrument-specific variations. Future research should focus on hybrid modeling strategies that integrate computed chemical shifts and couplings with curated experimental data sets through transfer learning or domain adaptation techniques. Such approaches are likely to improve model generalizability, reduce overfitting to synthetic data artifacts, and enable more reliable spectral prediction across novel compound classes. The unique ability to train on accurate, scalable, and interpretable computational data solidifies NMR’s position at the forefront of data-driven chemical analysis.

## 6 Quantum Utility in NMR Simulations: Progress, Prospects, and Challenges

Quantum computing has emerged as a compelling paradigm for simulating NMR-relevant phenomena, particularly as classical methods encounter exponential scaling barriers.^454^ Algorithms such as the Quantum Phase Estimation (QPE)^455,456^ and Variational Quantum Eigensolver (VQE)^457^ have been deployed to simulate spin Hamiltonians and molecular electronic structures central to NMR interpretation.^458^ Notably, Burov et al. (2024)^454^ demonstrated the first high-field NMR spectral simulations using gate-based quantum processors. By encoding NMR-relevant Hamiltonians comprising chemical shift and scalar coupling terms into a digitally simulated spin model using first-order Trotterization, and executing the circuit on IBM’s 127-qubit quantum processor (Eagle r3), gate-based quantum simulations of liquid-state, high-field NMR spectra were performed for molecular systems containing up to 11 proton spins (approximately 47 atoms).^454^ In this study, spectral features of biologically relevant molecules, including ATP and 4-methylvaleric acid, were reproduced with qualitative agreement to experimental 400 MHz high-field NMR data. Compressed sensing techniques and built-in circuit-level error mitigation were applied to improve spectral resolution and reduce sampling cost. This work, conducted by Burov et al., represents a foundational demonstration of quantum simulation of liquid-state NMR using NISQ processors,^459^ establishing a critical benchmark for advancing quantum utility in spectroscopic applications.^458^ The integration of dynamic circuit compilation with scalable gate-based execution has provided a practical route for modeling complex spin systems that are increasingly challenging for classical algorithms. Complementary efforts by HQS Quantum Simulations^460^ under the EU-funded NextNMR initiative^459,461^ are building hybrid quantum-classical platforms for structure verification and spectral deconvolution in complex biological matrices. Merz et al. have made significant strides in extending the applicability of Sample-based Quantum Diagonalization (SQD)^462–465^ to chemically relevant molecular systems, establishing a robust foundation for its future deployment in quantum NMR simulations. The SQD algorithm, originally developed in collaboration with IBM quantum researchers, was designed to deliver Full CI-level chemical accuracy through measurement-based diagonalization strategies. Quantum-accurate SQD simulations were demonstrated for supramolecular interactions,^466^ validating the method against classical reference data. Fragment-based Density Matrix Embedding Theory (DMET) was integrated with SQD^467^ to enable the simulation of large molecular systems using quantum processors. Implicit solvation effects were incorporated through IEF-PCM, allowing the quantum simulation of solvated polar molecules.^468^ These advances demonstrate a clear path toward the utility of quantum processors in the simulation of nontrivial systems relevant to spectroscopy, catalysis, and drug discovery. Collectively, these efforts signal that quantum-enhanced NMR, once aspirational, has entered the proof-of-concept stage with steadily expanding applicability to realistic chemical property computations.

Despite demonstrable progress, several hardware-centric limitations continue to constrain the scalability and accuracy of quantum NMR simulations. Noise and decoherence inherent in NISQ devices significantly degrade circuit fidelity, especially for simulations involving intricate spin–spin couplings and subtle chemical shift perturbations. As circuit depth increases often scaling quadratically with the number of spins accumulated gate errors and limited coherence times obscure fine spectral features such as J-splitting or long-range correlations. Qubit connectivity and gate calibration further influence simulation tractability, necessitating substantial transpilation overhead and introducing architecture-specific biases. Additionally, challenges in quantum state preparation for entangled spin networks, and imperfect readout processes, compound these issues. The reliance on low-order Trotter decompositions, while computationally efficient, restricts the accuracy of simulated Hamiltonian dynamics. The hardware-agnostic implementation of SQD, combined with the LUCJ ansatz,^469^ offers an alternative to traditional eigenvalue solvers by relying on quantum-sampled expectation values to reconstruct eigenstates. This makes SQD especially attractive for NMR simulations, where many-body correlations and sparse spectra dominate. Their protocol’s compatibility with shallow circuits aligns well with the capabilities of current quantum devices, mitigating several bottlenecks posed by depth-intensive approaches like QPE. These innovations underscore the necessity of algorithm–hardware codesign in overcoming persistent limitations.

Efforts to overcome these challenges have spurred a diverse portfolio of error mitigation and hybrid algorithmic strategies. IBM’s Qiskit Runtime supports circuit reparameterization, zero-noise extrapolation, and dynamic decoupling sequences that extend coherence and reduce operational infidelity. Virtual purification and probabilistic error cancellation have improved the accuracy of postprocessed observables, while tensor-network-inspired recompilation schemes reduce effective circuit depth. HQS Quantum Simulations has piloted digital-analog hybrid frameworks that exploit native hardware interactions to simulate NMR observables with lower resource demands. Merz et al.’s fragment-based SQD framework leverages DMET to partition large molecules, enabling scalable simulations of complex chemical systems by isolating active subspaces. This hybrid decomposition strategy facilitates accurate treatment of electron correlation and spin interactions relevant to NMR without the exponential resource demands of global treatment. Spin-squeezing protocols and advanced quantum state engineering techniques are also being explored to enhance spectral readout. As IBM’s roadmap^470,471^ continues with devices like Osprey, Heron, and the modular System Two, the path toward simulating significantly larger systems such as peptides or metalloprotein clusters becomes increasingly realizable.

Future trajectories in quantum NMR hinge on algorithm-hardware codesign, rigorous benchmarking, Hardware efficient Ansatz design and expanding molecular applicability. As illustrated by Burov et al.,^454^ scaling to 20-spin systems is plausible with near-term devices supporting 1000+ two-qubit gates. This opens the door to simulating spectroscopically rich systems such as small conformationally flexible biomolecules, catalytic centers, and reactive intermediates. IBM’s strategic focus on quantum advantage through accessible APIs, dynamic scheduling, and scalable hardware modularity continues to guide the field’s infrastructure. Coupling quantum simulations with classical machine learning models such as for shift prediction or spin topology inference may accelerate convergence toward utility. Recent development of the SQD formalism to include the IEF-PCM solvent model by Merz and colleagues,^468^ implemented via IBM’s Qiskit addon,^472^ provides a foundation for NMR-relevant simulations of solvated polar systems, a crucial step toward biologically and pharmaceutically meaningful applications. Meanwhile, NextNMR^461^ continues to bridge classical spectral processing with quantum backends, targeting applications in personalized medicine and mixture deconvolution. Ultimately, realizing robust quantum advantage in NMR will depend on sustained progress in error correction, coherence management, and hybrid algorithm design. The convergence of these efforts establishes a credible roadmap toward quantum-accelerated NMR spectroscopy.

## 7 Concluding Remarks

This review highlights the transformative impact of quantum chemical and machine learning methods in computational NMR, driving advancements in structural biology and related disciplines. Quantum mechanical approaches, such as density functional theory and coupled-cluster calculations, remain central to the accurate prediction of NMR parameters. These methodologies facilitate precise structure determination and validation across diverse chemical and biological systems. Additionally, QSAR models significantly contribute by enabling the quantitative interpretation of complex structure–activity relationships, further enhancing the understanding of molecular interactions and chemical properties.

Machine learning methods complement these quantum chemical approaches by leveraging extensive data sets to automate spectral assignments, enhance predictive accuracy, and efficiently analyze complex, multidimensional data. The synergy between QM precision, and ML-driven automation has substantially advanced computational NMR methods development, providing powerful tools capable of addressing increasingly intricate molecular systems. As computational techniques continue to evolve, integrating QM, and ML approaches promises significant improvements in the accuracy, scalability, and practical applicability of NMR. Such integration will shape future developments in structural and molecular sciences, bridging theoretical predictions and experimental validations to accelerate scientific discovery.

## Abbreviations

NMR: Nuclear Magnetic Resonance
DFT: Density Functional Theory
HF: Hartree–Fock
CC: Coupled-Cluster
QM: Quantum Mechanics
FF: Force Field
ML: Machine Learning
DL: Deep Learning
MD: Molecular Dynamics
AI: Artificial Intelligence
SMILES: Simplified Molecular Input Line Entry System
UCPT: Uncoupled perturbation treatment
PCM: Polarizable Continuum Models
GNN: Graph Neural Network
MPNN: Message Passing Neural Network
CNN: Convolutional Neural Network
RNN: Recurrent Neural Network
RL: Reinforcement Learning
VAE: Variational Autoencoder
GAN: Generative Adversarial Network
QSAR: Quantitative Structure–Activity Relationship
QSPR: Quantitative Structure–Property Relationship
QSDAR: Quantitative Spectrometric Data-Activity Relationship
HOMO: Highest Occupied Molecular Orbital
LUMO: Lowest Unoccupied Molecular Orbital
NOE: Nuclear Overhauser Effect
PCA: Principal Component Analysis
EPR: Electron Paramagnetic Resonance
GIAO: Gauge-Including Atomic Orbital
SOAP: Smooth Overlap of Atomic Positions
TL: Transfer Learning
XAI: Explainable Artificial Intelligence
MMFF: Merck Molecular Force Field
FC: Fermi Contact
SD: Spin-Dipole
PSO: Paramagnetic Spin–Orbit
DSO: Diamagnetic Spin–Orbit
SSCC: Spin–Spin Coupling Constant
CSD: Cambridge Structural Database
BMRB: Biological Magnetic Resonance Data Bank
NP-MRD: Natural Products Magnetic Resonance Database
GISSMO: Guided Ideographic Spin System Model Optimization
AZM: Azithromycin
ANN: Artificial Neural Network
MCSCF: Multiconfigurational self-consistent field methods
CASSCF: Complete active space self-consistent field
RASSCF: The restricted active space self-consistent field
FCI: Full configuration interaction theory
DLPNO: Domain-Based Local Pair Natural Orbital
CCSD­(T): Coupled-Cluster with Single, Double, and Perturbative Triple excitations
CP-DFT: Coupled-Perturbed Density Functional Theory
CSA: Chemical Shift Anisotropy
CASE: Computer-Assisted Structural Elucidation
CPCM: Conductor-like Polarizable Continuum Model
TMS: Tetramethylsilane
QM/MM: Quantum Mechanics/Molecular Mechanics
AF-QM/MM: Automated Fragmentation QM/MM
CHESHIRE: Chemical Shift Repository for Computed NMR Scaling Factors
SVM: Support Vector Machines
VAE: Variational Autoencoders
MAE: Mean Absolute Errors
RMS: Root Mean Square
GIPAW DFT: Gauge-Including Projector Augmented Wave Density Functional Theory
CoSCoSA: Comparative Structural Connectivity Spectra Analysis
CCS: Collisional Cross Sections
DP4-AI: Probability-Based NMR Structure Validation with AI
DP5: Standalone Probability Framework for NMR Structure Validation
ML-J-DP4: Machine Learning-enhanced J-DP4 Structural Assignment Method
CASCADE: ChemicAl Shift CAlculation with DEep learning
ExpNN: Experimental Neural Network
DFTNN: DFT-based Neural Network
LEGOLAS: neuraL nEtwork enGine fOr caLculating chemical Shifts
IMPRESSION: Intelligent Machine Prediction of Shift and Scalar Information of Nuclei
DMSO: Dimethyl Sulfoxide
QC: Quantum computing
QPE: Quantum Phase Estimation
VQE: Variational Quantum Eigensolver
SQD: Sample-based Quantum Diagonalization
DMET: Density Matrix Embedding Theory
